# Peripheral Cycloalkyl Functionalized Tetradentate Platinum(II) Phosphorescent Complex: Synthesis, Optical Tuning, and OLED Applications

**DOI:** 10.3390/ma18132942

**Published:** 2025-06-21

**Authors:** Giheon Park, Seon-jin Lee, Minsoo Kang, Wan Pyo Hong

**Affiliations:** 1 Department of Chemistry, Gachon University, 1342, Seongnam-daero, Sujeong-gu, Seongnma-si 13120, Gyeonggi-do, Republic of Korea; snlcpkh9701@gmail.com (G.P.); appley31@gachon.ac.kr (S.-j.L.); 2 SK Material JNC, 560, Dongtangiheung-ro, Hwaseong-si 18469, Gyeonggi-do, Republic of Korea

**Keywords:** OLED emitter, green phosphorescence, Pt(II) complex, tetradentate Pt complex

## Abstract

A tetradentate Pt(II) complex with a 5/6/6 structural backbone, Pt(PhPiPy-O-PytmCz), was synthesized by incorporating two distinct cycloalkyl groups. These structural modifications significantly enhanced the photoluminescence quantum yield and effectively increased the distance between molecules, thereby mitigating undesirable intermolecular interactions and triplet-state quenching. This strategic molecular design resulted in an external quantum efficiency of 11.5% at a wavelength of 539 nm and significantly enhanced operational lifetimes in green phosphorescent organic light-emitting diodes (OLEDs). These findings are expected to inspire the development of new green luminescent materials and innovative strategies in OLED technology.

## 1. Introduction

Organic light-emitting diodes (OLEDs) are highly energy-efficient and color-tunable, making them widely employed in flat-panel displays and promising candidates for next-generation displays and lighting technologies [1,2]. Currently, the OLED display industry balances stability and efficiency by utilizing a combination of fluorescent organic and phosphorescent metal emitters for blue, green, and red pixels. Blue pixels are typically fabricated employing fluorescent organic emitters owing to the operational lifetime limitations of phosphorescent blue emitters, despite their internal quantum efficiency (IQE) being constrained to a maximum of 62.5%, even with triplet–triplet annihilation (TTA) upconversion. In contrast, phosphorescent metal emitters, which are commercially utilized for green and red pixels, achieve an IQE of up to 100% [3,4,5,6].

In green OLEDs, phosphorescent Ir(III) complexes exhibit superior photophysical properties owing to their relatively short triplet exciton lifetimes and high photoluminescence quantum yields (PLQYs) [7,8]. However, the increasing demand for OLEDs has necessitated the development of alternative emitters to reduce their dependence on iridium, which is one of the scarcest elements in the Earth’s crust. Over the past decade, researchers have advanced pure organic thermally activated delayed-fluorescence (TADF) materials and phosphors based on more abundant metals such as Cu, Au, Pd, and Pt [9,10,11,12,13,14,15]. Some of these emitters now deliver device efficiencies comparable to or even surpassing those of Ir(III) complexes. Nevertheless, many of these alternatives have not demonstrated comparable operational stabilities [16].

Among the non-iridium luminescent materials, the development of Pt(II) complexes featuring phenyl-pyridine ligands is particularly noteworthy. While early studies focused on bidentate Pt (II) complexes such as (ppy)Pt(acac), their molecular configuration is influenced by the dsp^2^ hybrid orbitals adopted by the Pt(II) ion, resulting in a square-planar geometry [17]. This geometry increases the flexibility of bidentate Pt (II) complexes, enabling the excited state energy to dissipate through various nonradiative decay pathways, including molecular distortion and vibrational bonding modes. Consequently, the nonradiative decay rate is 4.5 times greater than the radiative decay rate in CH_2_Cl_2_ solution at room temperature.

To address these limitations, the development of more rigid and stable ligands is essential for creating efficient and stable Pt(II)-based phosphorescent emitters. Pt(II) complexes with judiciously designed phenylpyridyl-based tetradentate ligands, particularly those incorporating pyridyl-carbazole moieties, provide rational coordination sites for Pt(II) ions [18]. These tetradentate Pt(II) complexes simultaneously preserve the rigid square-planar configuration to enhance stability and reduce nonradiative decay pathways, thereby improving efficiency. Additionally, these features facilitate the synthesis of materials exhibiting high metallization yields. These complexes have demonstrated exceptional operational lifetimes, saturated emission colors, and high efficiencies. However, these advances have predominantly focused on blue phosphors thus far [19].

**Figure 1:**
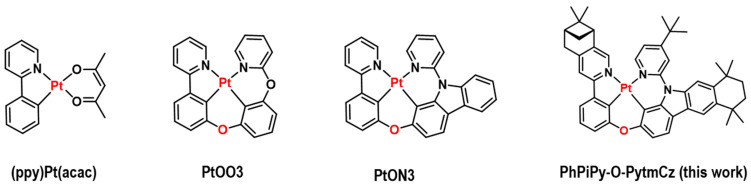
Molecular structures of (ppy)Pt(acac), PtOO3, PtON3, and Pt(PhPiPy-O-PytmCz).

Similar to blue phosphors, green phosphors based on tetradentate Pt(II) complexes exhibit more rigid molecular configurations and enhanced photophysical and chemical properties. For instance, the quantum yield (*ϕ*) of the phenoxyl-pyridine (popy)-based complex PtOO3 exceeds 80% in CH_2_Cl_2_ solution and approaches unity in a rigid PMMA matrix [20,21]. Further enhancement of *ϕ* to 100% in CH_2_Cl_2_ has been achieved by incorporating a more rigid carbazolyl-pyridine ancillary ligand, as demonstrated in the PtON3 complex [22]. These features make the PtON3 complex an ideal candidate for multifunctional optical materials, specifically OLED phosphors. Despite these advantages, the application of these tetradentate Pt green emitters, including the PtON3 complex-like emitter, remains limited, likely owing to their relatively long radiative decay lifetimes (typically > 4 μs). These extended lifetimes lead to emission saturation and annihilation effects under high exciton densities (Figure 1) [23]. Consequently, the long radiative decay lifetimes of these Pt complexes promote triplet–triplet annihilation (TTA) and/or triplet–polaron annihilation (TPA) processes, generating high-energy excited states that ultimately degrade the emitters [24,25].

Spatially bulky phosphorescent tetradentate Pt(II) complexes typically deliver outstanding electroluminescent performance, and their properties can be finely tuned by modifying the ligand backbone and/or peripheral substituents. These modifications shield the complexes from undesirable processes such as TTA and TPA [26,27,28]. Therefore, alkyl groups have been introduced at specific ligand positions to effectively reduce excited-state interactions. Numerous studies have demonstrated that the incorporation of alkyl moieties into luminescent cyclometalated complexes enhances the performance and stability of electroluminescent devices that use these complexes as light-emitting dopants. For example, Kim et al. improved the photochemical stability of a high-energy metal-centered triplet state by introducing bulky 3,5-di-*tert*-butyl-phenyl groups into the *N*-heterocyclic carbene (NHC) moiety of a tetradentate Pt(II) complex [29]. This modification also prevented undesirable host–guest interactions, contributing to a longer device lifetime and higher color purity. Similarly, She et al. incorporated *tert*-butyl groups into the tetradentate ligand framework, which improved the solubility and minimized intermolecular interactions that could otherwise induce TTA [30].

Despite significant advances, several limitations persist in tetradentate metal emitters, with design constraints limiting the overall performance of phosphorescent organic light-emitting diodes (PhOLEDs). In conventional NHC Pt(II) complexes, bulky groups—such as tert-butyl, mesityl, and 2,6-diisopropylphenyl—are typically employed to control the intermolecular packing; however, their inherent flexibility can increase the vibrational modes. In contrast, incorporating bulky cycloalkyl groups into tetradentate Pt(II) complexes can effectively restrict vibrational motion, suppress additional vibrations, and simultaneously enhance steric bulkiness [31].

Understanding the correlation between structural modifications and the tunable photophysical properties of Pt(II) complexes bearing fused cycloalkyl ligands is essential. Replacing the conventional *tert*-butyl substituent on carbazole with a tetramethylcyclohexyl (tmCy) unit fused to the carbazole ring markedly suppresses intermolecular interactions and vibrational modes, thereby enhancing the PLQY of the Pt(II) complexes. Additionally, pinene—a naturally occurring chiral group—can be incorporated into the pyridine moiety via a Kröhnke-type reaction. This modification suppresses noncovalent Pt⋯Pt and/or π-π interactions. A specifically designed complex, Pt(PhPiPy-O-PytmCz), is predicted by Density Functional Theory calculations to exhibit a Pt⋯Pt distance of 5.20 Å, significantly larger than the 4.0 Å observed in Pt(ppy) complexes [32,33]. Combined with its high PLQY, these results indicate that Pt(PhPiPy-O-PytmCz) is suitable as a green phosphor in PhOLEDs, delivering pure green emission with Commission Internationale de l’Éclairage (CIE) coordinates of (0.419, 0.550) and achieving a maximum external quantum efficiency (EQE_max_) of 11.4%.

## 2. Materials and Methods

### 2.1. Materials and Instrumentation

The reactions were monitored via thin-layer chromatography (TLC) utilizing Kieselgel 60 F254 silica gel plates. Flash chromatography was performed over silica gel 60, 230–400 mesh, utilizing the designated solvents. ^1^H and ^13^C NMR spectra were recorded on a Jeol 500 MHz spectrometer at room temperature (NMR; JNM-ECZ500R, Jeol Ltd., Tokyo, Japan) serviced by the Center for Bionano Materials Research at Gachon University (Seongnam, Republic of Korea). UV-vis absorption spectra were recorded employing a UV-1900 spectrometer (Shimadzu, Kyoto, Tokyo). Photoluminescence (PL) spectra were recorded on a Hitachi F-7100 fluorescence spectrophotometer and an Edinburgh FS5 Spectrofluorometer. Steady-state PL spectra and time-resolved PL decays in solution were recorded at 298 K for emission studies. Transient PL decay curves were obtained employing a time-correlated single-photon counting (TCSPC) Edinburgh FS5 spectrometer (Edinburgh Instruments Ltd., Livingston, UK). Transient spectra were collected for the prompt part and delayed part from a 365 nm picosecond pulsed LED. PL quantum yields were recorded utilizing an integrating sphere coupled to an Edinburgh FS5 under ambient conditions. Differential scanning calorimetry (DSC) was performed on a TA DSC Q2000 (TA Instruments, New Castle, DE, USA) at a heating rate of 10 °C min^−1^ under nitrogen. Thermogravimetric analysis (TGA) was performed on a TA SDT 650 instrument (TA Instruments, DE, USA) at a heating rate of 10 °C min^−1^ under nitrogen. The temperature at 5% weight loss was used as the decomposition temperature (T_d_). Cyclic voltammetry (CV) was performed on a WPG100e (WonATech, Seoul, Republic of Korea) instrument at room temperature with ferrocenium-ferrocene (Fc^+^/Fc) as the internal standard. Oxidative scans were performed using 0.1 M n-Bu_4_NPF_6_ (TBAPF_6_) in deoxygenated dichloromethane as the supporting electrolyte. Cyclic voltammograms were obtained at a scan rate of 0.1 V s^−1^.

The indium thin oxide (ITO)-coated glass, with a sheet resistance of 30 Ω/sq and thickness of 150 nm, was cleaned in an ultrasonic bath. Before the organic layer was deposited, the ITO-coated glass was dried in a convection oven at 120 °C for 10 min. Subsequently, the ITO-coated glass was subjected to O_2_ plasma treatment for 2 min at 2 × 10^−2^ Torr and 150 W. For the electroluminescence (EL) devices, all organic layers were deposited under the same vacuum conditions at a rate of 1 Å/s to cover an area of 4 mm^2^. The LiF and Al layers were continuously deposited under vacuum conditions. The current–voltage–luminance (J–V–L) properties of the fabricated EL devices were measured employing a Keithley 2400 electrometer (Tektronix, Cleveland, OH, USA) and the light intensities were measured employing a Minolta CS-1000A (Konica Minolta, Tokyo, Japan). The devices were stored in a glove box to maintain their stability against moisture and air.

### 2.2. Synthesis

#### 2.2.1. Synthesis of (6*R*,8*R*)-3-(3-Methoxyphenyl)-7,7-dimethyl-5,6,7,8-tetrahydro-6,8-methanoisoquinoline, (**W1**)

Pyridine (5 mL) and iodine (9.29 g, 36.62 mmol, 1.1 equiv) were added to a 100 mL 2-neck round-bottom flask. The mixture was stirred at room temperature for 1 h, after which 3′-methoxyacetophenone (4.57 mL, 33.29 mmol) was added. The reaction mixture was heated to 120 °C for 2 h. Upon completion, the resulting salt was washed with a mixture of hexane and ethyl acetate and filtered. The obtained salt (12.78 g) was isolated in a 98.34% yield. The corresponding product (11.62 g, 32.74 mmol) was added to a 2-neck round-bottom flask, followed by the addition of ethanol (70 mL). The mixture was stirred at room temperature for 30 min, after which ammonium acetate (6.31 g, 81.85 mmol, 2.5 equiv) was added. R-Myrtenal (5.48 mL, 36.01 mmol, 1.1 equiv) was then added, and the reaction mixture was refluxed for 16 h under N_2_. After cooling to room temperature, the reaction mixture was transferred to a separatory funnel and washed with brine and ethyl acetate. The organic and aqueous layers were separated and the extraction was repeated three times for each layer. The combined organic layers were dried over anhydrous MgSO_4_, filtered, and concentrated under reduced pressure. The crude product was purified via column chromatography utilizing a mixture of ethyl acetate and hexane (5% ethyl acetate in hexane; 2 L total). (6*R*, 8*R*)-3-(3-methoxyphenyl)-7, 7-dimethyl-5, 6, 7, 8-tetrahydro-6, 8-methanoisoquinoline (5.69 g, 20.05 mmol) was obtained as a brown, jelly like material in a 61.23% yield. ^1^H NMR (500 MHz, CDCl_3_) δ 8.21 (d, *J* = 0.7 Hz, 1H), 7.56 (dd, *J* = 2.6, 1.6 Hz, 1H), 7.54–7.47 (m, 2H), 7.35 (t, *J* = 7.9 Hz, 1H), 6.93 (ddd, *J* = 8.2, 2.6, 1.0 Hz, 1H), 3.88 (s, 3H), 3.01 (d, *J* = 2.8 Hz, 2H), 2.85 (t, *J* = 5.5 Hz, 1H), 2.70 (dt, *J* = 9.6, 5.8 Hz, 1H), 2.31 (tt, *J* = 5.8, 2.8 Hz, 1H), 1.42 (s, 3H), 1.26 (d, *J* = 1.7 Hz, 1H), 0.66 (s, 3H).

#### 2.2.2. 3-((6*R*,8*R*)-7,7-Dimethyl-5,6,7,8-tetrahydro-6,8-methanoisoquinolin-3-yl)phenol, (**W**)

(6*R*,8*R*)-3-(3-methoxyphenyl)-7,7-dimethyl-5,6,7,8-tetrahydro-6,8-meth-anoisoquinoline (5.69 g, 20.05 mmol) was added to a 2-neck round-bottom flask, then dichloromethane (10 mL) was added. The mixture was stirred at room temperature for 1 h, cooled to 0 °C, and then boron tribromide (20 mL) was slowly added. The reaction mixture was then stirred overnight. The reaction was quenched with water, followed by the addition of sodium bicarbonate and dichloromethane. The organic layer was separated, dried over anhydrous MgSO_4_, filtered, and concentrated under reduced pressure. The crude product was then washed with hexane and filtered. The product was obtained as a white powder in 98.67% yield. ^1^H NMR (500 MHz, CDCl_3_) δ 8.38 (s, 1H), 7.75 (s, 1H), 7.41–7.32 (m, 2H), 7.03 (d, *J* = 7.6 Hz, 1H), 3.50 (s, 1H), 3.22 (s, 2H), 3.00 (t, *J* = 5.4 Hz, 1H), 2.84 (dt, *J* = 10.9, 5.7 Hz, 1H), 2.48–2.38 (m, 1H), 1.47 (s, 3H), 1.29 (s, 1H), 0.69 (s, 3H); HRMS (ESI, *m*/*z*): calculated for C18H20NO, 266.1545; found, 266.1545.

#### 2.2.3. 3-Bromo-7,7,10,10-tetramethyl-7,8,9,10-tetrahydro-5*H*-benzo[*b*]carbazole, (**E1**)

2-Bromo-9*H*-carbazole (2.0 g, 8.16 mmol) and 2,5-dichloro-2,5-dimethylhexane (1.78 g, 9.80 mmol) were added to a 100 mL 2-neck round-bottom flask, then 1,2-dichloroethane (50 mL) was added. The mixture was stirred at room temperature for 30 min, and then aluminum chloride (0.65 g, 4.90 mmol) was added. The reaction mixture was refluxed overnight. After cooling, the reaction mixture was quenched with a sodium bicarbonate solution and water, and the organic layer was extracted with dichloromethane. The combined organic layers were dried over anhydrous MgSO_4_, filtered, and concentrated under reduced pressure. The residue was washed with pentane to obtain 3-bromo-7, 7, 10, 10-tetramethyl-7, 8, 9, 10-tetrahydro-5*H*-benzo [*b*] carbazole (2.6 g, 7.32 mmol) as a white solid in an 89.68% yield. ^1^H NMR (500 MHz, CDCl_3_) δ 7.96 (d, *J* = 0.7 Hz, 1H), 7.87 (dd, *J* = 8.1, 0.6 Hz, 1H), 7.84 (s, 1H), 7.51 (d, *J* = 1.7 Hz, 1H), 7.35 (s, 1H), 7.29 (ddd, *J* = 8.3, 1.7, 0.6 Hz, 1H), 1.80–1.74 (m, 4H), 1.40 (s, 6H), 1.37 (s, 6H); HRMS (ESI, *m*/*z*): calculated for C20H23NBr, 356.1014; found, 356.1012.

#### 2.2.4. 3-Bromo-5-(4-(*tert*-butyl)pyridin-2-yl)-7,7,10,10-tetramethyl-7,8,9,10-tetrahydro-5*H*-benzo[*b*]carbazole, (**E**)

3-bromo-7,7,10,10-tetramethyl-7,8,9,10-tetrahydro-5*H*-benzo[*b*]carbazole (7.22 g, 20.32 mmol) and 2-bromo-4-tert-butylpyridine (4 mL, 22.35 mmol, 1.1 equiv) were added to a 2-neck round-bottom flask. 1,4-Dioxane (50 mL) was added to the flask, and the mixture was stirred at room temperature for 40 min while degassing. Potassium phosphate (50.8 mmol, 2.5 equiv) was then added, and the reaction was stirred for an additional 30 min at room temperature. Afterwards, trans-amine (0.7 equiv) and copper(I) iodide (0.5 equiv) were added, and the reaction mixture was refluxed overnight. After cooling, the mixture was transferred into a separatory funnel and washed with brine and ethyl acetate. The organic and aqueous layers were separated and the extraction was repeated twice for each layer. The combined organic layers were dried over anhydrous MgSO_4_, filtered, and concentrated under reduced pressure. The crude product was purified via column chromatography utilizing a mixture of ethyl acetate and hexane (5% ethyl acetate in hexane, 3 L total) to obtain a white powder (6.4 g, 16.92 mmol) in an 83.31% yield. ^1^H NMR (500 MHz, CDCl_3_) δ 8.63 (dd, *J* = 5.3, 0.7 Hz, 1H), 8.11 (d, *J* = 1.7 Hz, 1H), 8.01 (s, 1H), 7.91 (d, *J* = 8.3 Hz, 1H), 7.64 (s, 1H), 7.60 (dd, *J* = 1.8, 0.7 Hz, 1H), 7.37 (dd, *J* = 8.3, 1.7 Hz, 1H), 7.30 (dd, *J* = 5.3, 1.7 Hz, 1H), 1.78 (s, 4H), 1.42 (s, 6H), 1.41 (s, 9H), 1.34 (s, 6H); HRMS (ESI, m/z): calculated for C29H34N2Br, 489.1905; found, 489.1904.

#### 2.2.5. 5-(4-(*tert*-Butyl)pyridin-2-yl)-3-(3-((6*R*,8*R*)-7,7-dimethyl-5,6,7,8-tetrahydro-6,8-methanoisoquinolin-3-yl)phenoxy)-7,7,10,10-tetramethyl-7,8,9,10-tetrahydro-5*H*-benzo[*b*]carbazole, (**P1**)

(6*R*,8*R*)-7,7-dimethyl-5,6,7,8-tetrahydro-6,8-methanoisoquin-olin-3-yl)phenol (3.33 g, 12.55 mmol) and 3-bromo-5-(4-(*tert*-butyl)pyridin-2-yl)-7,7,10,10-tetramethyl-7,8,9,10- tetrahydro-5*H*-benzo[*b*]carbazole (4.91 g, 13.81 mmol) were added to a 100 mL 2-neck round-bottom flask, then DMSO (30 mL) was added. The mixture was stirred at room temperature for 20 min while degassing. Potassium phosphate (6.66 g, 31.38 mmol) was then added, and the mixture was stirred for another 20 min at room temperature. Picolinic acid (0.31 g, 2.51 mmol) and copper(I) iodide (0.03 g, 0.13 mmol) were then added, and the reaction mixture was heated to a bath temperature of 140 °C and stirred overnight. After cooling, the reaction mixture was quenched with brine and water and the organic layer was separated. The combined organic layers were dried over anhydrous MgSO_4_, filtered, and concentrated under reduced pressure. Each layer was subjected to three workups. The crude product was purified via column chromatography utilizing a mixture of ethyl acetate and hexane (10% ethyl acetate in hexane, 4 L total), yielding a yellow jelly-like product (4.9 g, 7.27 mmol, 57.98% yield). ^1^H NMR (500 MHz, CDCl_3_) δ 8.57 (d, *J* = 5.4 Hz, 1H), 8.20 (s, 1H), 8.06 (d, *J* = 8.8 Hz, 2H), 7.78 (s, 1H), 7.76 (dt, *J* = 7.9, 1.3 Hz, 1H), 7.73 (t, *J* = 2.1 Hz, 1H), 7.70 (d, *J* = 2.1 Hz, 1H), 7.62 (d, *J* = 1.7 Hz, 1H), 7.48 (s, 1H), 7.41 (t, *J* = 7.9 Hz, 1H), 7.22 (dd, *J* = 5.3, 1.7 Hz, 1H), 7.11 (ddd, *J* = 8.1, 2.5, 1.0 Hz, 1H), 7.07 (dd, *J* = 8.4, 2.1 Hz, 1H), 2.96 (d, *J* = 2.8 Hz, 2H), 2.82 (t, *J* = 5.5 Hz, 1H), 2.66 (dt, *J* = 9.4, 5.8 Hz, 1H), 2.25 (tt, *J* = 5.8, 2.9 Hz, 1H), 2.12 (s, 2H), 1.80 (s, 3H), 1.46 (s, 6H), 1.39 (d, *J* = 2.4 Hz, 10H), 1.34 (s, 9H), 0.64 (s, 3H); HRMS (FAB^+^, *m*/*z*): [M+H]^+^ calculated for C47H51N3O, 674.4109; found, 674.4113.

#### 2.2.6. PhPiPy-O-PytmCz

**P1** (4.45 g, 6.60 mmol) and acetic acid (30 mL) were added to a 100 mL 2-neck round-bottom flask. The mixture was degassed for 30 min, followed by the addition of tetrabutylammonium bromide (2.12 g, 6.60 mmol). The mixture was stirred at room temperature for 30 min, after which potassium tetrachloroplatinate(II) (K_2_PtCl_4_, 3.01 g, 7.26 mmol) was added. The reaction mixture was then refluxed for 36 h. After cooling, the reaction mixture was quenched with sodium bicarbonate and extracted with ethyl acetate. The organic layer was separated, dried over anhydrous MgSO_4_, filtered, and concentrated under reduced pressure. The crude product was purified via column chromatography utilizing a mixture of ethyl acetate and hexane (10% ethyl acetate in hexane, 4 L total), yielding a yellow solid (2.27 g, 1.97 mmol in a 29.81% yield. ^1^H NMR (500 MHz, CDCl_3_) δ 8.94 (d, *J* = 6.3 Hz, 1H), 8.25 (s, 1H), 8.17 (d, J = 2.0 Hz, 1H), 7.92 (s, 1H), 7.81 (s, 1H), 7.75–7.70 (m, 2H), 7.48 (dd, *J* = 7.2, 1.4 Hz, 1H), 7.30 (d, *J* = 8.2 Hz, 1H), 7.26–7.17 (m, 2H), 7.11 (dd, *J* = 6.3, 2.0 Hz, 1H), 3.11 (s, 2H), 2.87 (t, *J* = 5.4 Hz, 1H), 2.77 (dt, *J* = 10.8, 5.8 Hz, 1H), 2.37 (dt, *J* = 5.9, 3.0 Hz, 1H), 1.80 (s, 4H), 1.47–1.42 (m, 12H), 1.38 (s, 9H), 1.36 (s, 4H), 1.26 (s, 1H), 0.68 (s, 2H); 3C NMR (126 MHz, CDCl3) δ 163.78, 162.82, 153.28, 153.17, 150.54, 149.74, 147.83, 147.12, 143.82, 143.43, 142.13, 141.25, 139.85, 137.42, 127.36, 124.44, 124.31, 119.34, 117.70, 117.65, 117.49, 116.60, 116.15, 115.27, 112.74, 112.67, 112.13, 100.23, 44.79, 39.98, 39.63, 35.67, 35.44, 35.39, 35.06, 34.69, 33.33, 32.89, 32.72, 32.59, 32.00, 31.69, 30.38, 26.00, 22.76, 21.56, 14.24; HRMS (FAB^+^, *m*/*z*): [M+H]^+^ calculated for C_47_H_49_N_3_O, 867.3590; found, 867.3598. Anal. Calcd. for C47H49N3OPt: C 65.11, H 5.70, N 4.85. Found: C 65.08, H 5.74, N 4.90.

## 3. Results and Discussion

### 3.1. Molecular Design, Synthesis, and Characterization

**Figure 2:**
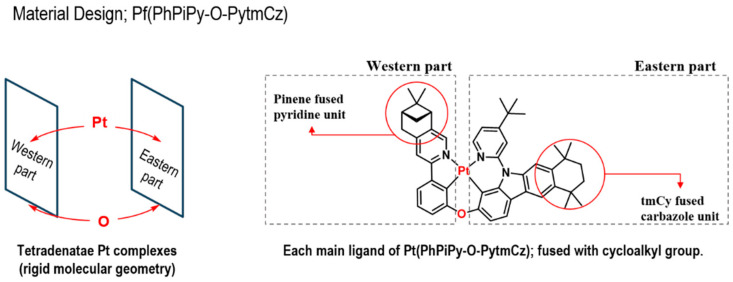
Material design of Pt(PhPiPy-O-PytmCz).

The principal quenching pathway in Pt-OLEDs involves Dexter-type intermolecular electron exchange between long-lived triplet excitons, which markedly reduces device efficiency [30]. Mitigating this process requires increasing the spacing between emitter molecules to suppress exciton quenching. Moreover, square-planar Pt(II) complexes are susceptible to weak Pt···Pt interactions via dz^2^-orbital overlap, making sterically bulky molecular design essential for maintaining photoluminescence in the solid state. To this end, we introduced bulky pinene and tmCy substituents into the tetradentate ligand, enlarging the distance between neighboring complexes, minimizing orbital overlap, suppressing Dexter transfer, and thus preserving triplet emission and markedly enhancing device performance. In this context, we investigate a new green phosphorescent emitter, Pt(PhPiPy-O-PytmCz), featuring a tetradentate framework that links a phenyl-pyridine (ppy) moiety on the west to a carbazole-pyridine fragment on the east. These ligands were selected for their strong π-back-bonding capability and ability to maintain an overall neutral charge in the complex. Both rigid ligands were connected to the Pt center via *O*-bridged coordination. To further rigidify the complex and limit aggregation, the ppy ligand was fused with a pinene motif, whereas the carbazole-pyridine fragment contained a bulky tmCy substituent (Figure 2). These sterically demanding pinene and tmCy groups effectively suppressed intermolecular interactions, thereby minimizing exciton self-quenching in the tetradentate Pt(II) complex.

**Scheme 1:**
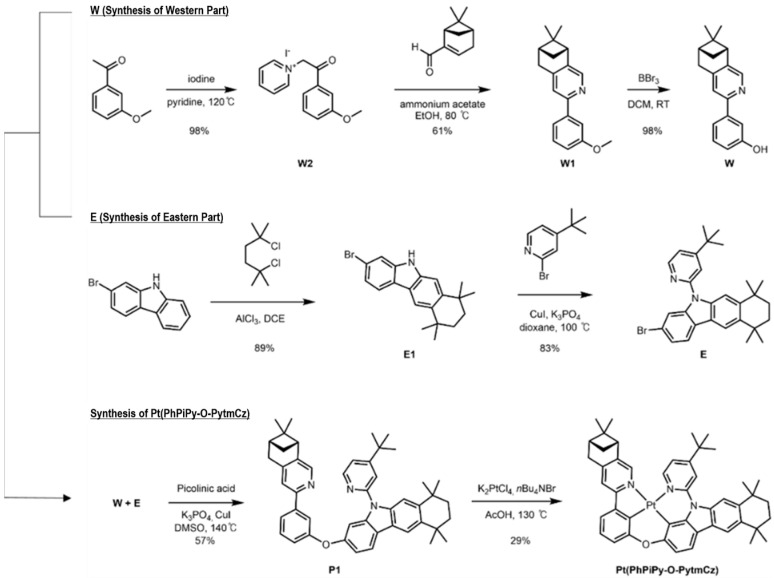
Synthesis of Pt(PhPiPy-O-PytmCz).

The pinene-2-phenyl pyridine ligand (**W1**) was synthesized in two steps, commencing with commercially available 3′-methoxyacetophenone, which was demethylated utilizing BBr_3_ to produce the western fragment (**W**). For the eastern fragment, a tmCy group was introduced into 2-bromo-9*H*-carbazole via Friedel–Crafts alkylation with 2,5-dichloro-2,5-dimethylhexane. Subsequently, 3-bromo-5-(4-(tert-butyl)pyridin-2-yl)-7,7,10,10-tetramethyl-7,8,9,10-tetrahydro-5H-benzo[b]carbazole (**E**) was synthesized via Ullmann coupling utilizing 2-bromo-4-(tert-butyl)pyridine and copper(I) iodide. Alcohol intermediates **W** and **E** were then coupled via C–O bond formation mediated by copper(I) iodide, resulting in the formation of P1. Finally,

Pt(PhPiPy-O-PytmCz) was synthesized in a 30% yield via metalation with potassium tetrachloroplatinate(II) and a base. The synthetic procedures are outlined in Scheme 1, with detailed descriptions provided in the Supporting Information.

### 3.2. Photophysical Properties

**Figure 3:**
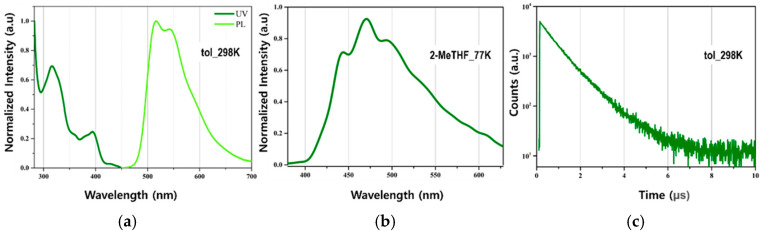
(**a**) UV-vis and PL spectra, (**b**) low-temperature PL spectra, and (**c**) TRPL curves of Pt(PhPiPy-O-PytmCz).

The UV-vis absorption and PL spectra were analyzed in 1.0 × 10^−5^ M toluene solutions at room temperature to investigate the photophysical properties of Pt(PhPiPy-O-PytmCz). As shown in Figure 3a, absorption bands at 317 nm were primarily attributed to the cyclometallic ligand-centered (^1^LC) transition. Bands in the >395 nm region were assigned to mixed singlet metal-to-ligand charge transfer (^1^MLCT), ^3^π-π*, and ^3^MLCT transitions, which were facilitated by the strong spin-orbit coupling of the Pt atom [34]. Additionally, the energy gap for Pt(PhPiPy-O-PytmCz), calculated from the onset of the UV-vis spectrum, was 2.56 eV.

Upon photoexcitation, Pt(PhPiPy-O-PytmCz) exhibited green emission with peaks at approximately 516 nm and a shoulder at approximately 544 nm. In contrast, Pt(ppy), as reported by She et al., displayed a featureless and broad green emission exhibiting a peak at 520 nm. These differences in the emission profiles clearly indicate distinct lowest-emitting triplet characteristics. Considering the structured PL spectra of Pt(PhPiPy-O-PytmCz), the ligand-centered ^3^π-π* state likely contributes more significantly to the emission than PtON3, whereas the broad and structureless PL spectrum of PtON3 suggests that its emission predominantly originates from the charge-transfer (CT) state. This observation aligns with the distribution of the lowest unoccupied molecular orbital (LUMO) in Theoretical Calculations. In PtON3, the LUMO was localized across both pyridine segments, whereas in Pt(PhPiPy-O-PytmCz), it was partially distributed on the pyridine moiety of the eastern segment. PL emission spectra were also measured at 77 K in tetrahydrofuran (THF) at a concentration of 1.0 × 10^−5^ M. At this low temperature, a rigidochromic shift of 470 nm was observed, resulting in a highly structured emission accompanied by a shorter emission lifetime (Figure 3b). The sharp and structured emission of Pt(PhPiPy-O-PytmCz), along with its extended lifetime, indicate that the ligand-centered states dominated in the frozen solution.

PLQY and transient PL (TrPL) measurements were performed to assess the potential of Pt(PhPiPy-O-PytmCz). Notably, the PLQY value was determined to be 83%, and a short exciton lifetime of 2.14 μs was observed in the TrPL measurements. Accordingly, the radiative rate constant (*k*_r_) and nonradiative rate constant (*k*_nr_) were calculated using the PLQY and exciton lifetime values via the simple relation (1) [26,29].

(1) $$PLQY=\frac{k_{r}^{T}}{k_{r}^{T}+k_{nr}^{T}}=\frac{k_{r}^{T}}{k_{d}}$$

$k_{r}^{T}$: triplet radiative rate constant, $k_{nr}^{T}$: triplet non-radiative rate constant, $k_{d}$: decay rate constant.

The *k*_r_ value was calculated as 3.9 × 10^5^ s^−1^, and the relatively low *k*_nr_ value of 7.9 × 10^4^ s^−1^ is attributed to the rigidity of the tetradentate ligand. This rigidity results from the incorporation of two cycloalkyl groups, namely the pinene and tmCy units, which mitigated the detrimental effects associated with the nonradiative decay rate constant (*k*_nr_) by effectively restricting the critical vibrational motions of the flexible alkyl chains. These structural characteristics collectively contributed to the suppression of nonradiative decay. Table 1 summarizes the PLQY, exciton lifetimes, and photophysical parameters of Pt(PhPiPy-O-PytmCz).

### 3.3. Theoretical Calculations

**Figure 4:**
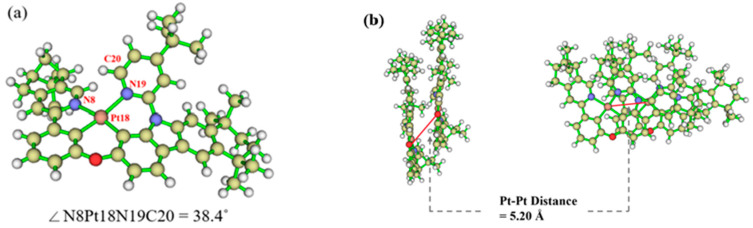
(**a**) Optimized structure of Pt(PhPiPy-O-PytmCz). (**b**) Dimer characteristics of Pt(PhPiPy-O-PytmCz). Dimer side view (**left**) and top view (**right**).

Managing the intermolecular interactions is challenging owing to the inherently planar geometry of the main backbone. Therefore, the geometry of the tetradentate phosphor and its molecular interactions must be carefully optimized. In this context, Pt(PhPiPy-O-PytmCz) was strategically designed to address these challenges: (1) The dihedral angle between the two pyridine units, represented as ∠N8Pt18N19C20, was calculated, employing Gaussian methods, to be 38.4° in Pt(PhPiPy-O-PytmCz), compared with 23.3° in PtON3 (Figure 4a) [35]. This increased dihedral angle represents sufficient distortion to prevent planar geometry and reduce potential intermolecular overlap [36]. (2) Pt(PhPiPy-O-PytmCz) was designed, incorporating bulky blocking groups: pinene on the western part and tmCy in conjunction with *tert*-butyl on the eastern part of the tetradentate ligand. These groups effectively increase intermolecular separation, enabling only weak intermolecular interactions. While *tert*-butyl is a relatively small blocking group, pinene and tmCy are significantly bulkier and more effective in preventing dz^2^ orbital overlap through the bulky moieties. This design resulted in a nearest Pt-Pt distance of 5.20 Å (Figure 4b). Consequently, the typical dimer formation observed in planar Pt(II) emitters was efficiently suppressed by the twisted conformation and strategic incorporation of the three bulky moieties in Pt(PhPiPy-O-PytmCz).

**Figure 5:**
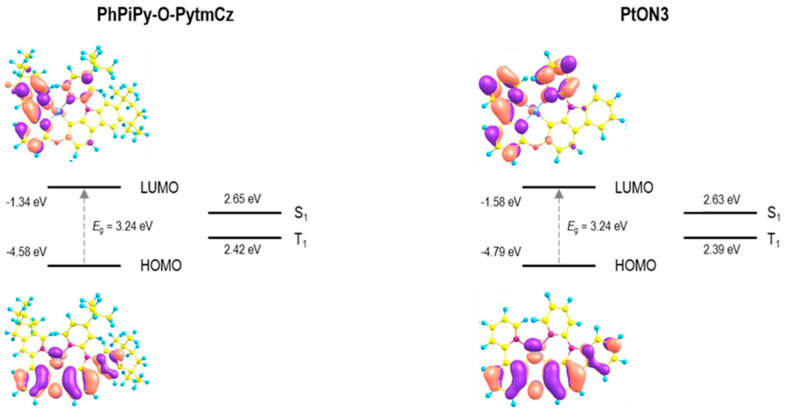
Isodensity surface plot and HOMO/LUMO levels of Pt(PhPiPy-O-PytmCz) and PtON3.

Ground-state geometry optimization and time-dependent density functional theory (TD-DFT) calculations were performed using the B3LYP functional comprising the genECP/LANL2DZ basis set for Pt and 6-31G(d,p) basis set for C, N, O, and H in the Gaussian 16 program. The highest occupied molecular orbital (HOMO) was primarily localized on the Pt central metal, phenyl group, and carbazole unit of the molecule. The lowest unoccupied molecular orbital (LUMO) was primarily distributed over the ppy and pyridine units on the eastern part of the molecule. However, the distribution in the pyridine unit was less pronounced than that in PtON3 (Figure 5). This reduced distribution is attributed to the *tert*-butyl group, which destabilized the LUMO of the pyridine component. Consequently, a shallower calculated LUMO energy level (−1.34 eV) was observed for Pt(PhPiPy-O-PytmCz) compared with that of PtON3 (−1.58 eV).

TD-DFT calculations were performed to clarify the nature of the relevant excited states, focusing on the energy gap, excitation characteristics, and molecular frontier orbitals of Pt(PhPiPy-O-PytmCz). The low-lying S_0_→T_1_ transition in Pt(PhPiPy-O-PytmCz) revealed that the T_1_ state exhibited a mixed character of metal-to-ligand charge transfer (^3^MLCT), intraligand charge transfer (ILCT), and ^3^π-π* transitions (Table S1). These findings indicate that the emission originates from a combination of locally excited (LE) and charge-transfer (CT) states, aligning with the photophysical measurements obtained in solution. Furthermore, a relatively high MLCT component (11.2%) was observed, which is critical for achieving a high PLQY and short excited-state lifetime [37,38].

### 3.4. Electroluminescence (EL) Properties

To determine the HOMO and LUMO energy levels and design the OLED architecture, the oxidation and reduction behavior of Pt(PhPiPy-O-PytmCz) were examined via cyclic voltammetry (CV). Measurements were conducted in dichloromethane (CH_2_Cl_2_) and dimethylformamide (DMF) solutions for anodic and cathodic scans, respectively, at room temperature. The resulting CV curves and corresponding electrochemical data are presented in the Supporting Information, indicating that Pt(PhPiPy-O-PytmCz) undergoes a reversible oxidation process. The onset potential of the first oxidation wave ($E_{ox}^{onset}$) was used to calculate the HOMO energy level using the equation *E_HOMO_* = −*e*($E_{ox}^{onset}$ + 4.4) eV [39], validating a HOMO level of −4.91 eV. Similarly, the LUMO energy level was determined from the onset potential of the first reduction wave ($E_{red}^{onset}$) using the corresponding equation *E*_LUMO_ = −*e*($E_{red}^{onset}$ + 4.4) eV. The deep LUMO energy level of −1.76 eV, which was attributed to its two pyridine units, aligns well with the calculated trends. These findings demonstrate that the pyridine ligands significantly influence the LUMO energy levels, enabling the fine-tuning of the energy band gap and electronic properties of Pt(PhPiPy-O-PytmCz), thereby making it a viable candidate as a green phosphor in OLEDs.

The electroluminescent (EL) properties of Pt(PhPiPy-O-PytmCz) were studied by fabricating OLEDs with the following structure: ITO/N-([1,1′-biphenyl]-4-yl)-9,9-dimethyl-N-(4-(9-phenyl-9H-carbazol-3-yl)phenyl)-9H-fluoren-2-amine (BCFN): 2-(7-dicyanomethylene-1,3,4,5,6,8,9,10-octafluoro-7H-pyrene-2-ylidene)-malononitrile (NDP-9) (10 nm, 3 wt %)/BCFN (90 nm)/9-(3-(triphenylsilyl)phenyl)-9H-3,9′-bicarbazole (SiCzCz) (5 nm)/SiCzCz: 9,9′-(6-(3-(triphenylsilyl)phenyl)-1,3,5-triazine-2,4-diyl)bis(9H-carbazole) (SiTrzCz2):Pt(PhPiPy-O-PytmCz) (7:3, 1/2/3/5 wt %, 30 nm)/2-phenyl-4,6-bis(3-(triphenylsilyl)phenyl)-1,3,5-triazine (mSiTrz) (5 nm)/mSiTrz: 8-quinolinolato lithium (Liq) (2:1, 30 nm)/LiF (1 nm)/Al (150 nm) (Figure S13). In these optimized devices, BCFN and SiCzCz served as the hole-transporting and electron-blocking layers, respectively, whereas mSiTrz functioned as both the exciton-blocking and electron-transporting layers. SiCzCz and SiTrzCz_2_ were selected as the hole- and electron-transporting hosts, respectively, to construct an exciplex-based co-host system for the emitter. This exciplex not only balances charge injection but, owing to its high triplet energy, also suppresses back energy transfer from the emitter to the host. Consequently, emission from PhPiPy-O-PytmCz is generated primarily through direct energy transfer from the exciplex to the dopant [40,41].

An analysis of the EL characteristics revealed that Pt(PhPiPy-O-PytmCz) exhibited a peak EL wavelength (EL_max_) at 539 nm with CIE coordinates of (0.419 and 0.550), confirming its suitability for practical green PhOLEDs. When the dopant concentration was increased from 3 to 5 wt %, EL_max_ remained at 539 nm; however, the shoulder band at ~650 nm intensified marginally, which was attributed to intermolecular interactions between the Pt(II) complexes. Nevertheless, shifts in the CIE coordinates were effectively suppressed by the sterically hindered spacers positioned on the eastern and western sides of the molecule.

**Figure 6:**
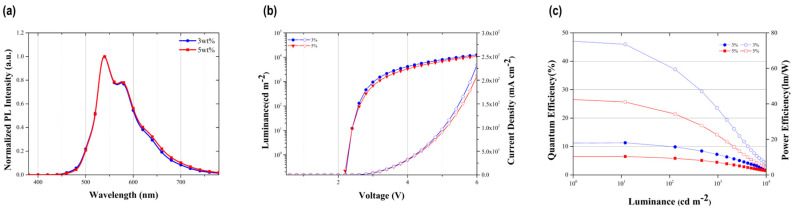
EL performance of doped OLED devices: (**a**) EL spectra, (**b**) current-density (J)–voltage (V)–luminance (L) characteristics, and (**c**) EQE vs. L curves.

Figure 6b presents the current-density–voltage–luminance (J–V–L) curves for the devices containing 3 and 5 wt % Pt(PhPiPy-O-PytmCz). At 3 wt % doping, the device achieved a maximum luminance (L_max_) of 12,532 cd m^−2^, peak current efficiency (CE_max_) of 35.08 cd A^−1^, peak power efficiency (PE_max_) of 48.03 lm W^−1^, and maximum external quantum efficiency (EQE_max_) of 11.3%. Even at 1000 cd m^−2^, it maintained a CE of 22.34 cd A^−1^, PE of 23.30 lm W^−1^, and EQE of 7.17%. The turn-on voltage was as low as 2.4 V, which is lower than those of previously reported green PhOLEDs, owing to the small hole/electron-injection barriers and relatively short triplet lifetime of Pt(PhPiPy-O-PytmCz). These results demonstrate the strong potential of Pt (PhPiPy-O-PytmCz) as a green emitter that combines a high EQE with excellent power efficiency [42].

Furthermore, lifetime measurements were performed at an initial luminance of 3000 cd m^−2^ on devices doped at 3, 5, and 7 wt %. As shown in Figure S15, the corresponding LT_90_ values (time for the luminance to fall to 90% of its initial value) are 25.6, 30.8, and 38.5 h, respectively, indicating that device lifetime is strongly dependent on the Pt(PhPiPy-O-PytmCz) concentration. Using the empirical relation LT_90_(L_1_) = LT_90_(L_0_) × (L_0_/L_1_)^1.7^, these values extrapolate to 165.7, 199.3, and 249.2 h at 1000 cd m^−2^ [43]. This performance places our Pt-OLEDs among the stable Pt-based devices reported to date (Figure S17), underscoring the intrinsic stability of Pt(PhPiPy-O-PytmCz).

## 4. Conclusions

We successfully introduced a tetradentate 5/6/6 structural backbone core that incorporates two unique cycloalkyl groups for the synthesis of Pt(PhPiPy-O-PytmCz), providing a direct and efficient synthetic pathway. These structural modifications significantly increased the intermolecular distance between molecules in the solid state, thereby suppressing intermolecular aggregation. This molecular design enhanced photophysical properties, including PLQY, sublimability, and solubility. In device applications, it reduced undesirable intermolecular interactions and minimized triplet-state quenching.

This strategic molecular design achieved a peak EQE value of 11.5% at a wavelength of 539 nm and, by increasing the doping ratio, significantly enhanced the operational lifetimes of green OLEDs. These findings open new avenues for optimizing emissive materials, ultimately improving the device efficiency and stability.

## Figures and Tables

**Table 1:** Photophysical properties and kinetic parameters of Pt(PhPiPy-O-PytmCz) in toluene solution.

	UV-vis ^[a]^ (nm)	λ_max_ ^[a]^ (nm)	FWHM ^[a]^ (nm)	*Φ* ^[b]^ (%)	*τ* ^[a]^ (μs)	*k*_r_ ^[c]^ (×10^5^ s^−1^)	*k*_nr_ ^[d]^ (×10^4^ s^−1^)	HOMO ^[e]^ (eV)	LUMO ^[e]^ (eV)
Pt(PhPiPy-O-PytmCz)	317, >420	516 (544)	88	83.5	2.14	3.9	7.9	−4.91	−1.76

[a] Toluene solution (5.0 × 10^−5^ M), r.t. [b] PLQY at 300 K measured in the integrating sphere under N_2_. [c] Radiative decay rate constant (*k*_r_ = PLQY/τ). [d] Non-radiative decay rate constant: *k*_nr_ = (1 − PLQY)/τ. [e] Calculated from cyclic voltammetry analysis.

## Data Availability

The original contributions presented in this study are included in the article/Supplementary Material. Further inquiries can be directed to the corresponding authors.

## Supplementary Materials

The following supporting information can be downloaded at https://www.mdpi.com/article/10.3390/ma18132942/s1.
