# Supplementary material for: Peripheral Cycloalkyl Functionalized Tetradentate Platinum(II) Phosphorescent Complex: Synthesis, Optical Tuning, and OLED Applications
Source: Materials (Basel). 2025 Jun 21;18(13):2942. doi: 10.3390/ma18132942 (PMC12250862; doi:10.3390/ma18132942)
Supplement: Supplementary file 1 [file materials-18-02942-s001.zip › materials-3652737-supplementary.pdf]

Peripheral Cycloalkyl Functionalized Tetradentate Platinum(II) Phosphorescent Complex: Synthesis, Optical Tuning, and OLED Applications

Giheon Park [1,†], Seon-jin Lee [1,†], Minsoo Kang [2,*] and Wan Pyo Hong [1,*]

[1] Department of Chemistry, Gachon University, 1342, Seongnam-daero, Sujeong-gu, Seongnma-si 13120, Gyeonggi-do, Republic of Korea; snlcpkh9701@gmail.com (G.P.); appley31@gachon.ac.kr (S.-j.L.)

[2] SK Material JNC, 560, Dongtangiheung-ro, Hwaseong-si 18469, Gyeonggi-do, Republic of Korea

* Correspondence: mskang2@sk.com (M.K.); wphongw@gachon.ac.kr (W.P.H.); Tel.: +82-31-750-8529

† These authors contributed equally to this work.

Page

1. Figure S1. 1H-NMR spectrum of compound (W1)……………………………………..……3

2. Figure S2. 1H-NMR spectrum of compound (W)…..…………………………………..…....3

3. Figure S3. 1H-NMR spectrum of compound (E1)……………………….……..……....4

4. Figure S4. 1H-NMR spectrum of compound (E)……………………….……………...…....4

5. Figure S5. 1H-NMR spectrum of compound (P1) ……………………………..……....5

6. Figure S6. 1H-NMR spectrum of compound (Pt(PhPiPy-O-PytmCz))……………..……....5

7. Figure S7. 13C-NMR spectrum of compound (Pt(PhPiPy-O-PytmCz))…………..……….6

8. Figure S8. PL spectra of Pt(PhPiPy-O-PytmCz) in different organic solvents…..……..……..6

9. Figure S9. Emission decay curves obtained at emission maximum for Pt(PhPiPy-O-PytmCz) in the toluene…………………………………………………………..…..……..………..7

10. Figure S10. Consecutive cyclic voltammetry (CV) curves for Pt(PhPiPy-O-PytmCz)……..7

11. Figure S11. Thermogravimetric analysis curves for Pt(PhPiPy-O-PytmCz)…...…….….......8

12. Figure S12. DSC thermograms of Pt(PhPiPy-O-PytmCz recorded under nitrogen at a heating rate of 10°C/min……………………………………………………………….........8

13. Figure S13. Energy diagram of OLEDs and the molecular structure of materials used for fabricating OLEDs…………………………………………………….…………...…....9

14. Figure S14. Luminance (L)–voltage (V) characteristics...…...………………………….…..9

15. Figure S15. Operational lifetimes of Pt(PhPiPy-O-PytmCz)……………………….………..10

16. Figure S16. EL performances of doped OLED devices ………………………..…………10

17. Figure S17. The operational lifetimes of representative green emitters……………………11

18. Figure S18. The selected frontier orbitals of Pt(PhPiPy-O-PytmCz) ………………………11

19. Figure S19. HR-MS of HR-MS of ((6R,8R)-7,7-dimethyl-5,6,7,8-tetrahydro-6,8-methanoisoquinolin-3-yl)phenol (W)……………………………………………………...12

20. Figure S20. HR-MS of HR-MS of 2.2.3. 3-bromo-5-(4-(tert-butyl)pyridin-2-yl)-7,7,10,10-tetramethyl-7,8,9,10- tetrahydro-5H-benzo[b]carbazole (E1)……………………...………12

21. Figure S21. HR-MS of HR-MS of 3-bromo-5-(4-(tert-butyl)pyridin-2-yl)-7,7,10,10-tetramethyl-7,8,9,10- tetrahydro-5H-benzo[b]carbazole (E)…………………………….…13

22. Figure S22. HR-MS of PhPiPy-O-PytmCz (P1)…………………………………………13

23. Figure S23. HR-MS of Pt(PhPiPy-O-PytmCz)…………………………………….……14

24. Table S1. Computed absorption wavelengths…………………………….…………..14

25. Table S2. Molecular orbital energies …………………………………………...……..15

26. Table S3. Cartesian coordinates of the ground state (S0) ……………………….……..15

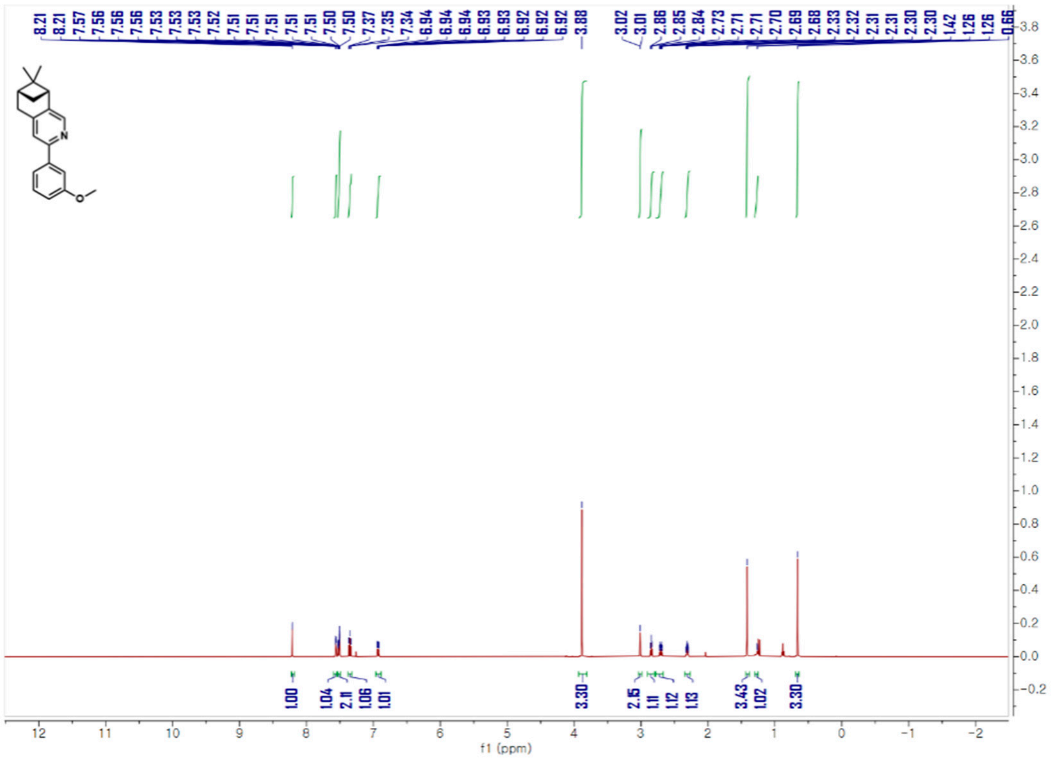

Figure S1. ^{1}H-NMR spectrum of compound (W1)

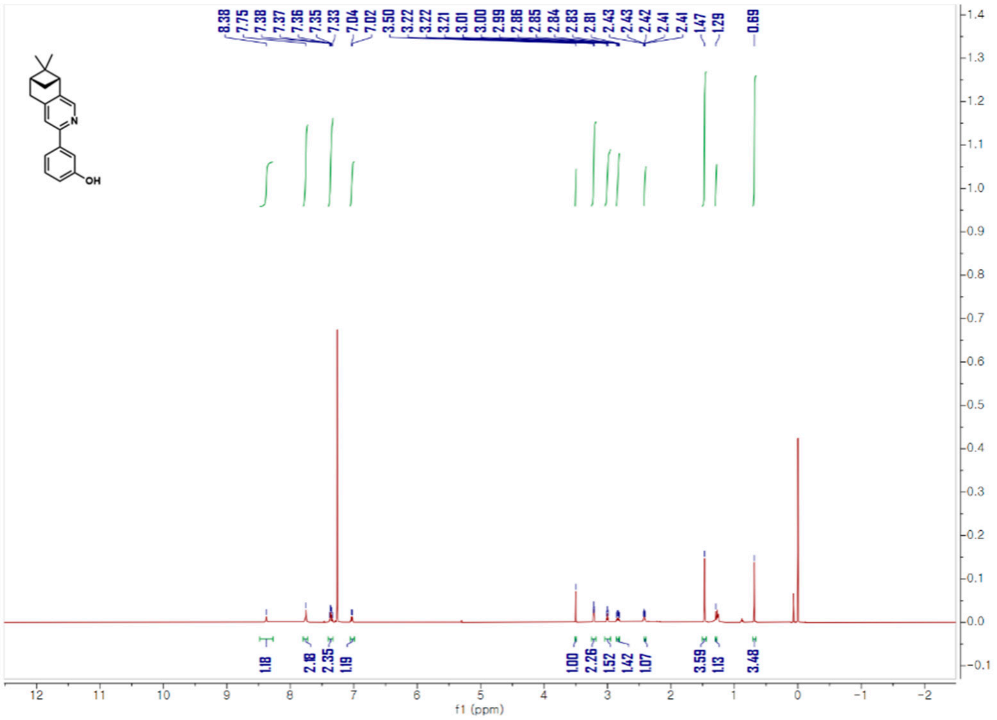

Figure S2. ¹H-NMR spectrum of compound (W)

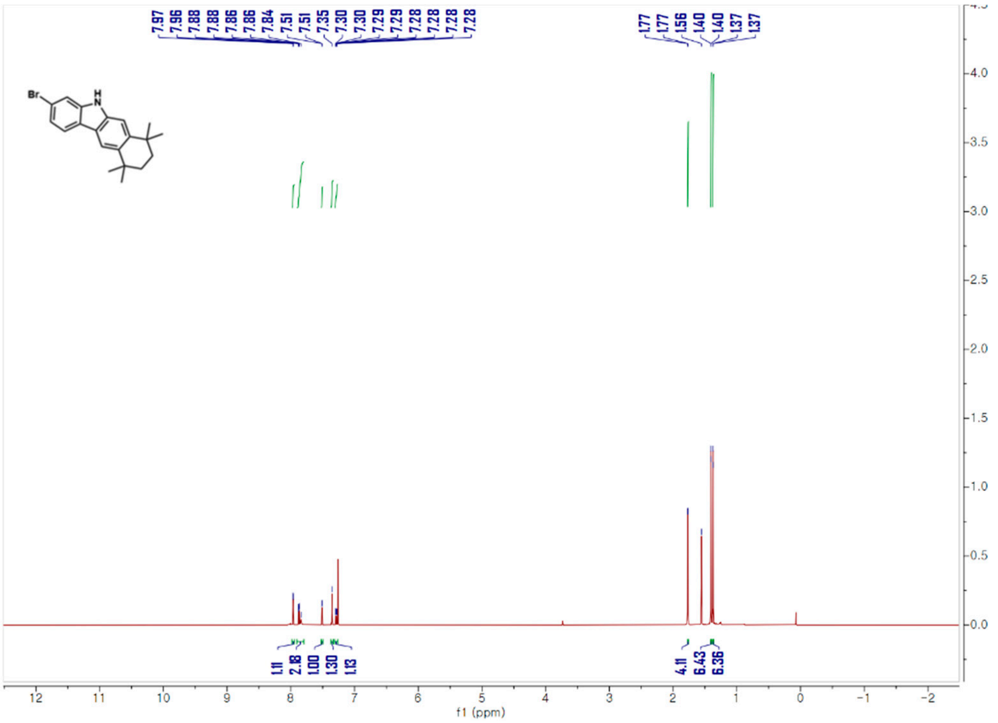

Figure S3. ¹H-NMR spectrum of compound (E1)

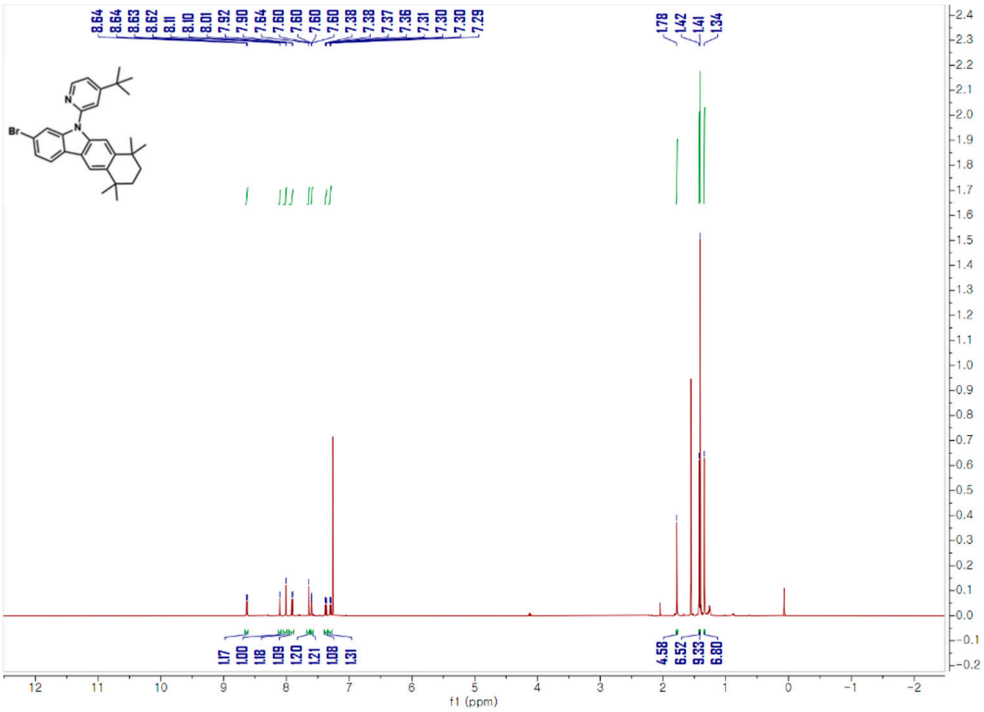

Figure S4. ¹H-NMR spectrum of compound (E)

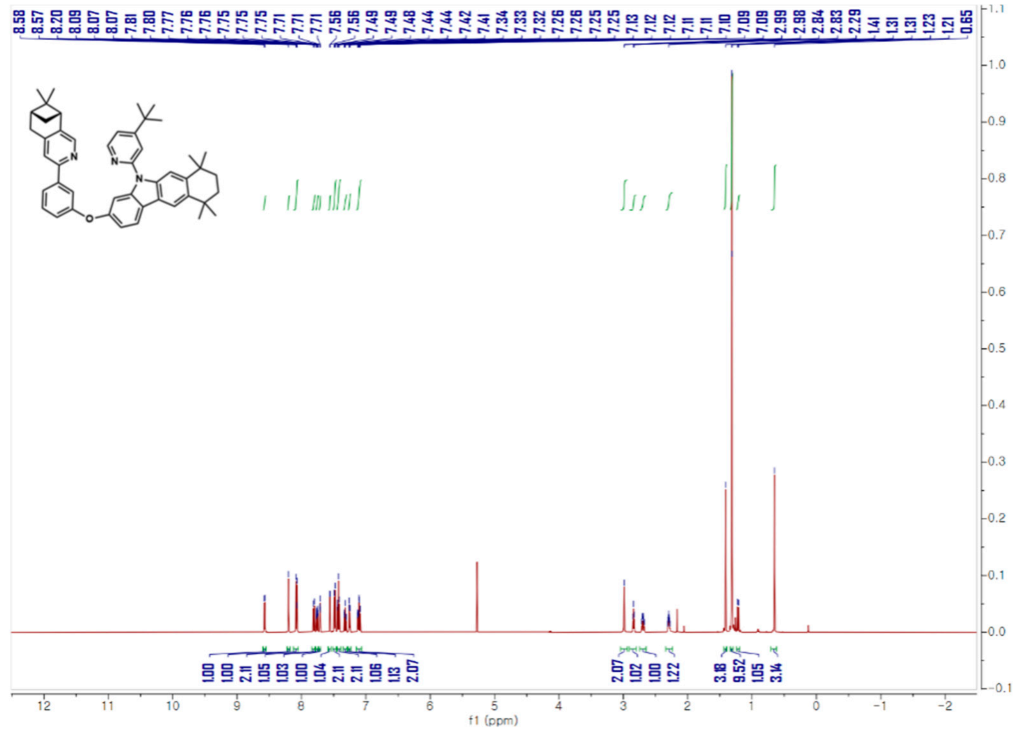

Figure S5. ^{1}H-NMR spectrum of compound (P1)

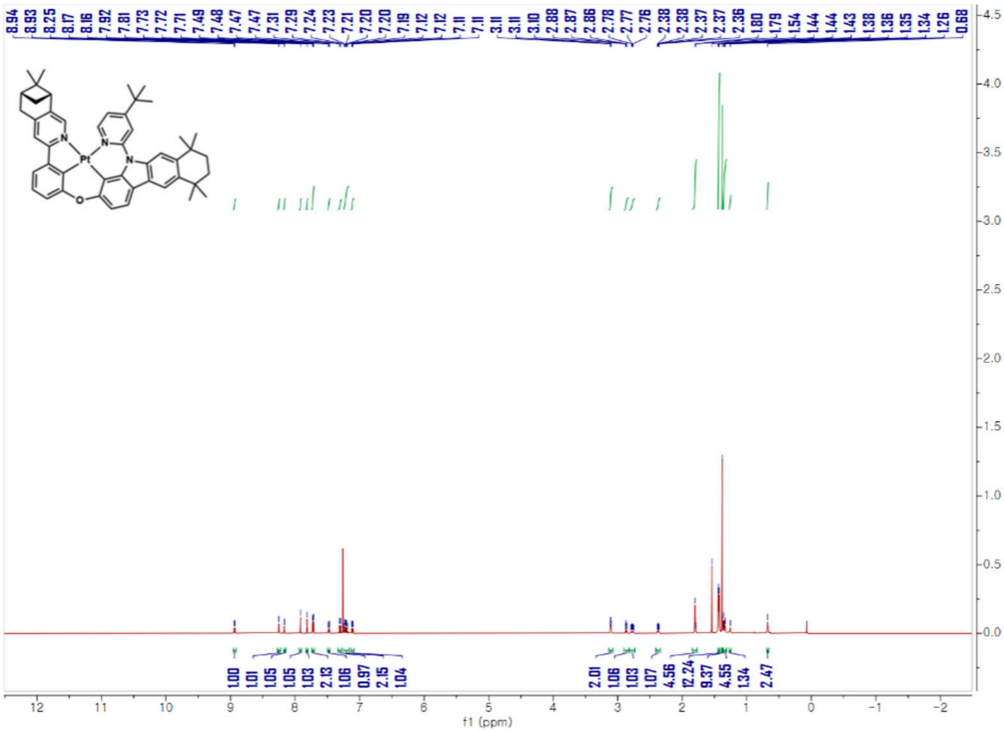

Figure S6. ^{1}H-NMR spectrum of compound (Pt(PhPiPy-O-PytmCz))

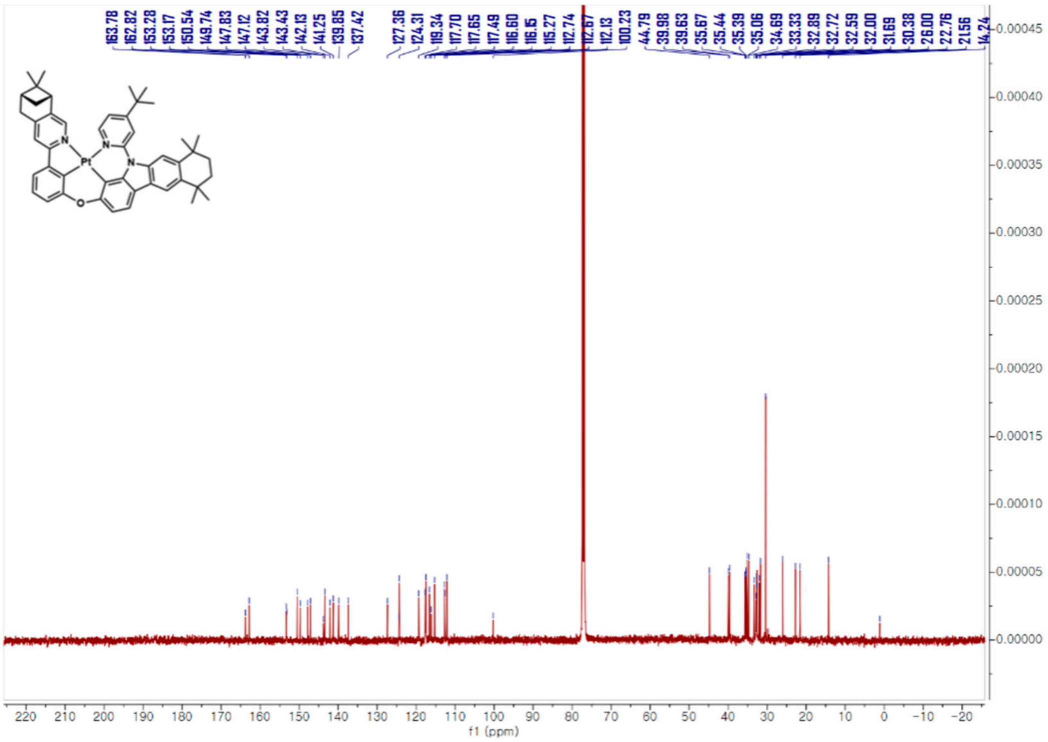

Figure S7. ^{13}C-NMR spectrum of compound (Pt(PhPiPy-O-PytmCz))

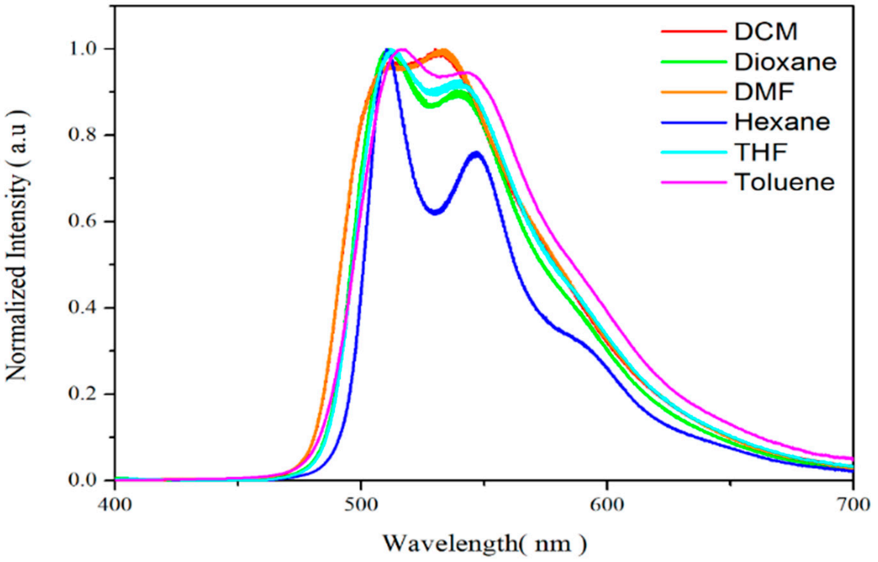

Figure S8. PL spectra of Pt(PhPiPy-O-PytmCz) (λex = 365 nm) in different organic solvents (5×10^{-5}M, DMF: dimethylformamide, THF: tetrahydrofuran, and DCM: dichloromethane).

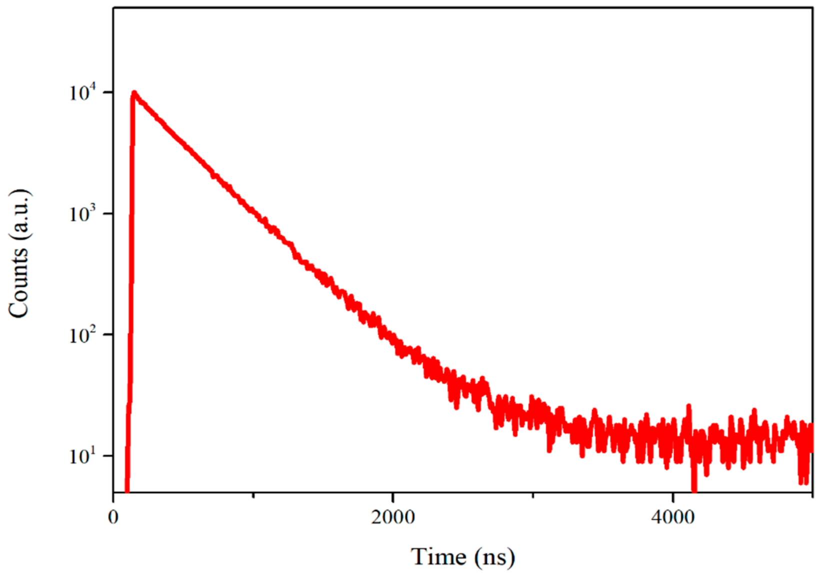

Figure S9. Emission decay curves obtained at emission maximum for Pt(PhPiPy-O-PytmCz) in the toluene.

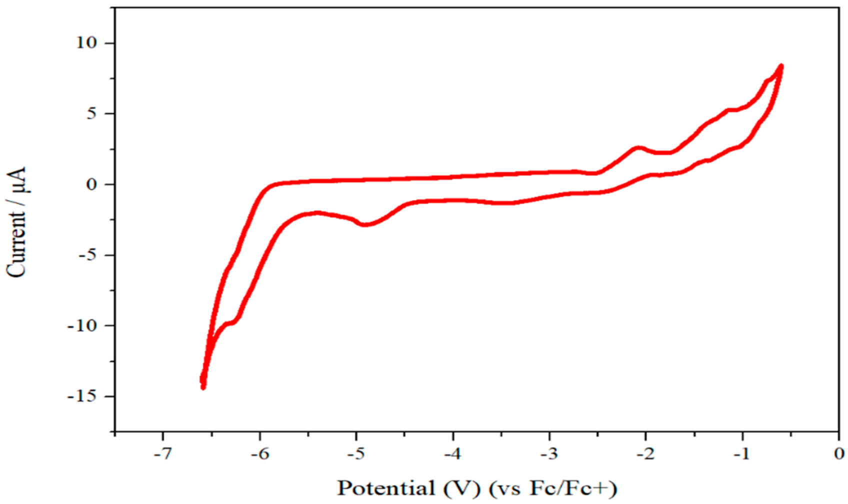

Figure S10. Consecutive cyclic voltammetry (CV) curves for Pt(PhPiPy-O-PytmCz).

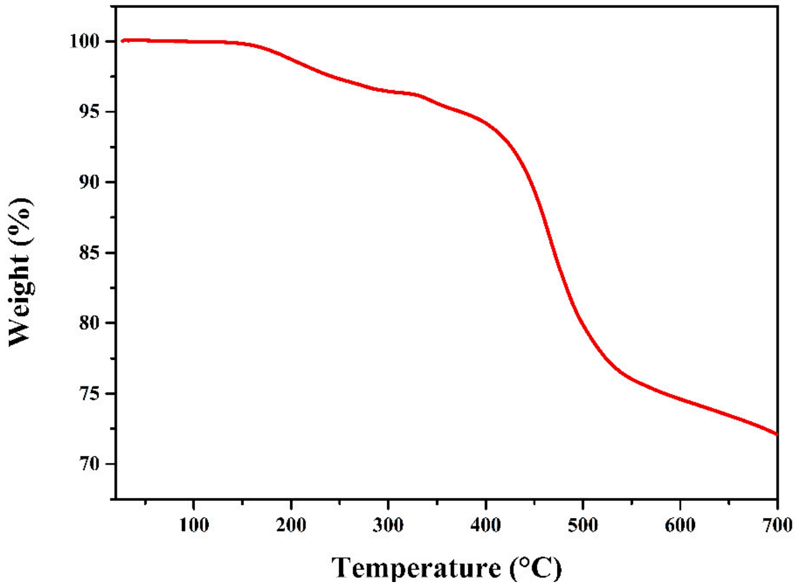

$T_d(1\%) = 190.9$ ℃, $T_d(5\%) = 373.9$ ℃

Figure S11. Thermogravimetric analysis curves for Pt(PhPiPy-O-PytmCz).

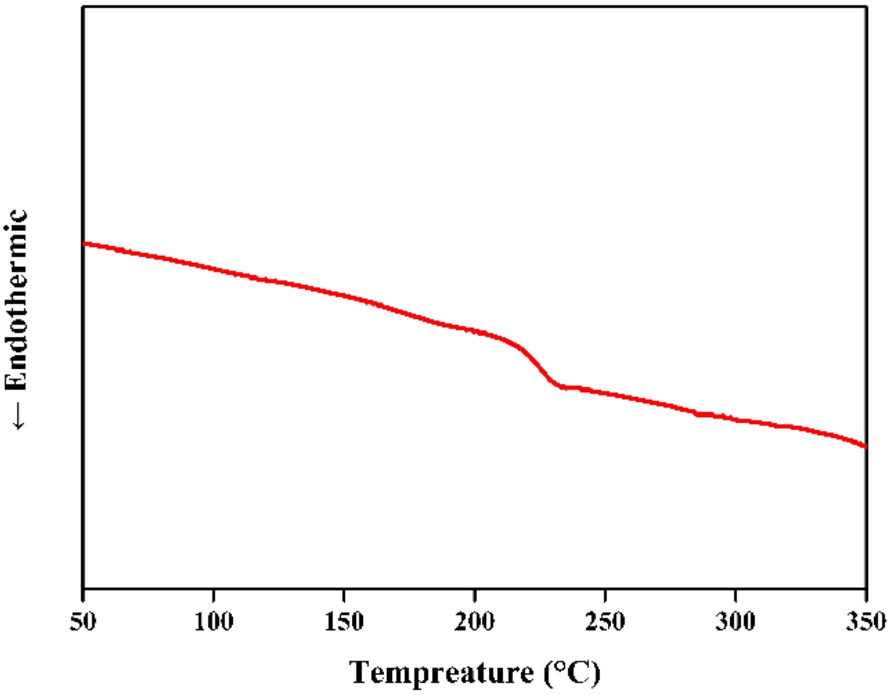

Figure S12. DSC thermograms of Pt(PhPiPy-O-PytmCz recorded under nitrogen at a heating rate of 10°C/min

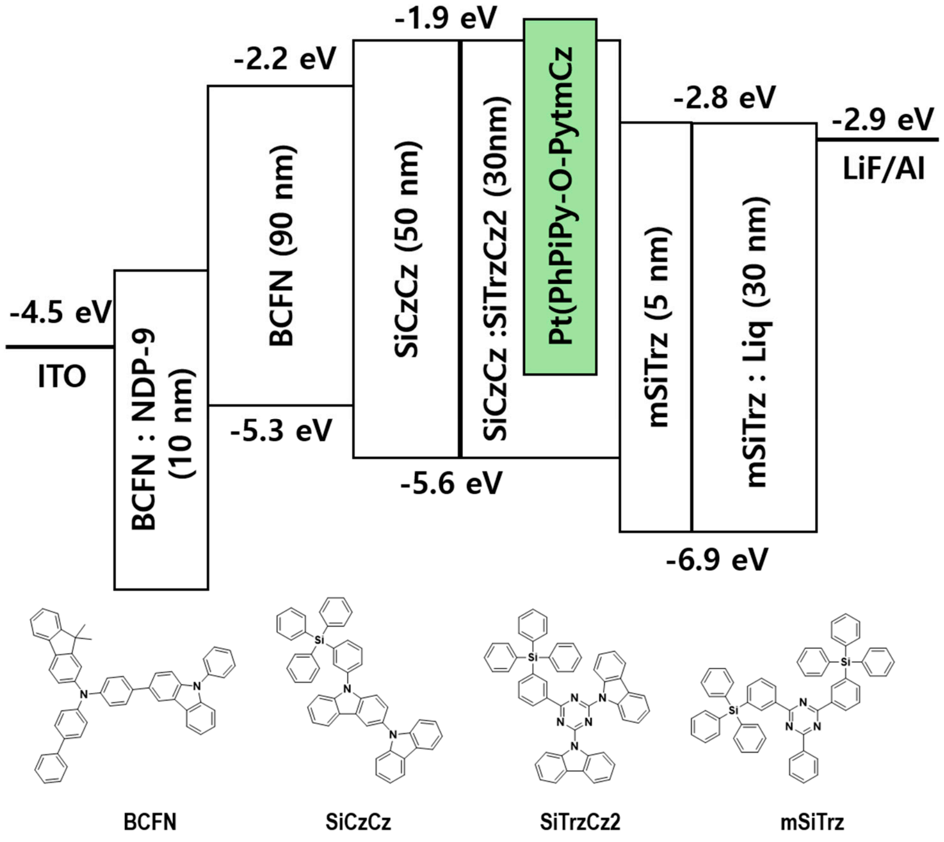

Figure S13. Energy diagram of OLEDs and the molecular structure of materials used for fabricating OLEDs.

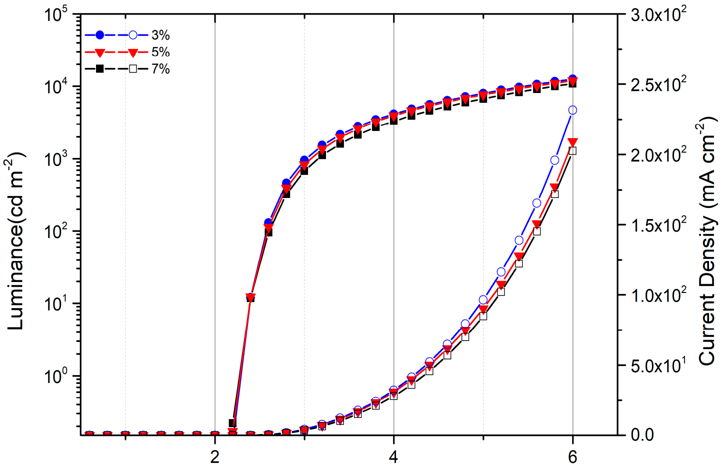

Figure S14. Luminance (L)–voltage (V) characteristics (the inset shows current density (J)–voltage (V) characteristics) for the OLEDs fabricated using Pt(PhPiPy-O-PytmCz) (3-7wt%) with a SiCzCz:SiTrCz2 mixed host.

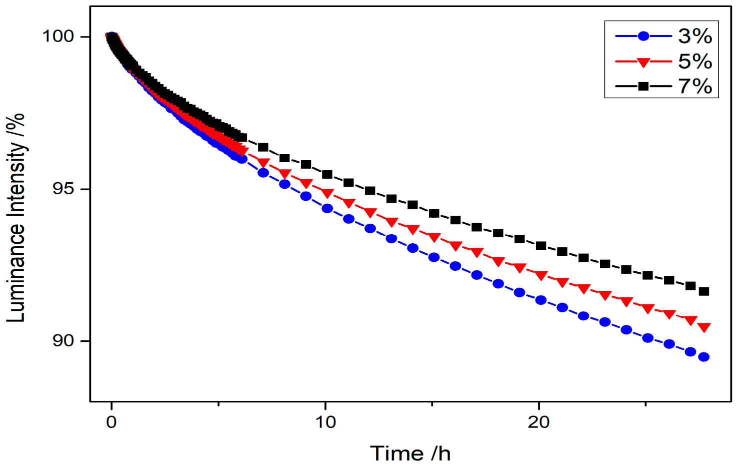

Figure S15. Operational lifetimes of Pt(PhPiPy-O-PytmCz)-based green phosphorescent OLEDs. Initial luminance = 3000 cd/m^2.

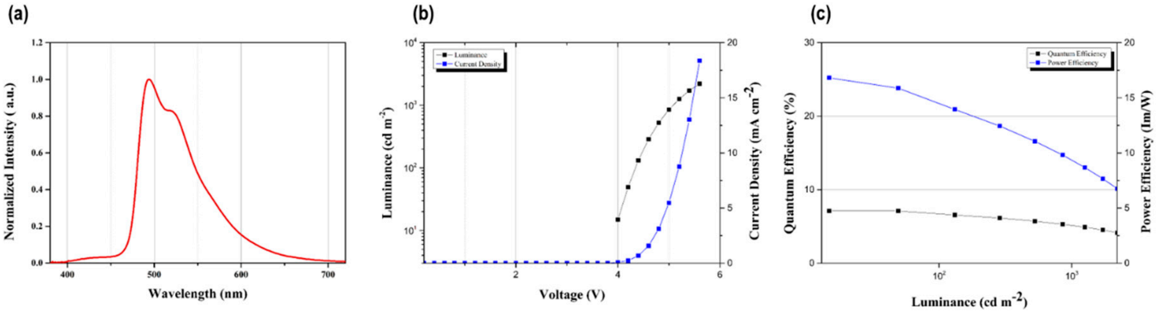

We tested 1,3-Bis(carbazol-9-yl)benzene (mCP), a commercially available host material, instead of SiCzCz/SiTrzCz$_2$ exciplex-type hosts. At 5 wt% doping, the device achieved a maximum luminance (L_{max}) of 2210 cd m^{-2}, peak current efficiency (CE_{max}) of 21.41 cd A^{-1}, peak power efficiency (PE_{max}) of 16.81 lm W^{-1}, and maximum external quantum efficiency (EQE_{max}) of 7.12%.

While the host differs slightly from mCP to 3,3′-Di(9H-carbazol-9-yl)-1,1′-biphenyl (mCBP), the EQE of Ir(ppy$_3$) was reported as 11.8% (*Sci. Rep.* **2013**, *3*, 2127).

Figure S16. EL performances of doped OLED devices: (a) EL spectra, (b) current density (J)-voltage (V)-luminance (L) characteristics, and (c) EQE vs L curves.

Emitter	PL	EL Performance					Reference
	λ [nm]	λ [nm]	EQE_{max} [%]	EQE_{1000} [%]	CIE (x,y)	Operation Lifetime	
Phosphorescence Emitters							
Pt(PhPiPy-O-PytmCz)	516	539	11.3	7.17	(0.41,0.55)	LT90 = 25.6 h @3000 cd/m^2	This Work
Pt-1	524	532	25.1	-	(0.25,0.67)	LT95 = 133 h @10000 cd/m^2	*Small* **2024**, 20, 2307393
Ir(TBF)$_2$(mppy)	527	531	27.1		(0.36,0.62)	LT90 =57.8 h @3000 cd/m^2	*Adv. Opt. Mater.* **2022**, *10*, 2201511
Ir(tpsp)$_2$(bpp)	528	-	27.9	27.8	(0.37,0.60)	LT50 =61892 h @100 cd/m^2	*J. Phys. Chem. C.* **2021**, *125*, 24671
(BzIPr)AuBN	511	513	24.8	24.6	(0.18,0.70)	LT60 =1210 h @1000 cd/m^2	*Angew. Chem. Int. Ed.* **2022**, *61*, e202213392
Fluorescence/ TADF Emitters							
DBN-NaPh-d	521	526	35.2	26.4	(0.21,0.74)	LT95 =45 h @1000 cd/m^2	*Adv. Sci.* **2025**, *37*, 2411610
tPhBODIPY	518	520	19	18.9	(0.26,0.67)	LT50=2947h @1000 cd/m^2	*Adv. Opt. Mater.* **2020**, *8*, 2000483
tPBn-BODIPY	531	532	19.5		(0.24,0.65)	LT50=2503h @1000 cd/m^2	*Angew. Chem. Int. Ed.* **2023**, *62*, e202312666
TCz-B	512	525	29.2	9.4	(0.16,0.71)	LT50=2h @1000 cd/m^2	*Angew. Chem. Int. Ed.* **2021**, *60*, 23142
BpIC-Cz	534	544	25.7	24.3	(0.34,0.64)	LT90=291.5h @1000 cd/m^2	*Chem. Eng. J.* **2024**, *481*, 148794

Figure S17. The operational lifetimes of representative green emitters.

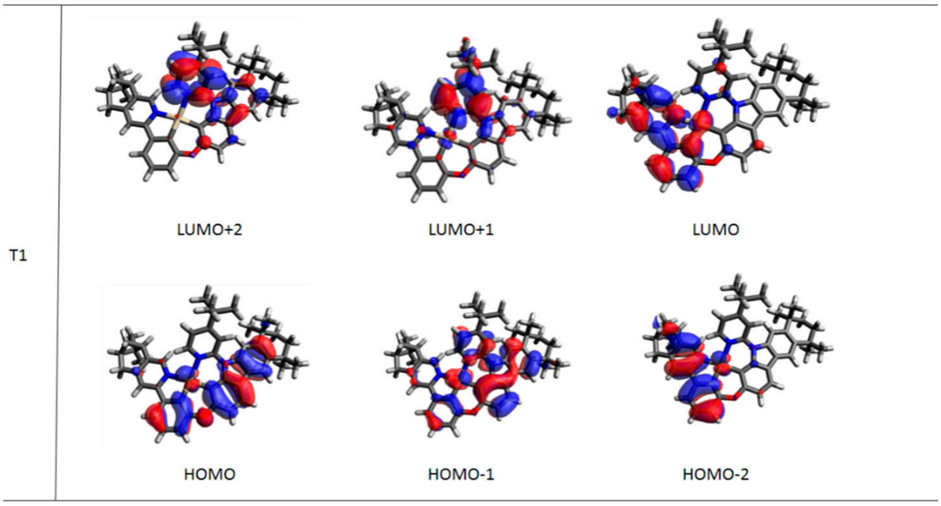

Figure S18. The selected frontier orbitals of Pt(PhPiPy-O-PytmCz) from PBE0 calculations (Isovalue = 0.04 a.u.) at the first triplet excited state (T1) optimized geometries in toluene.

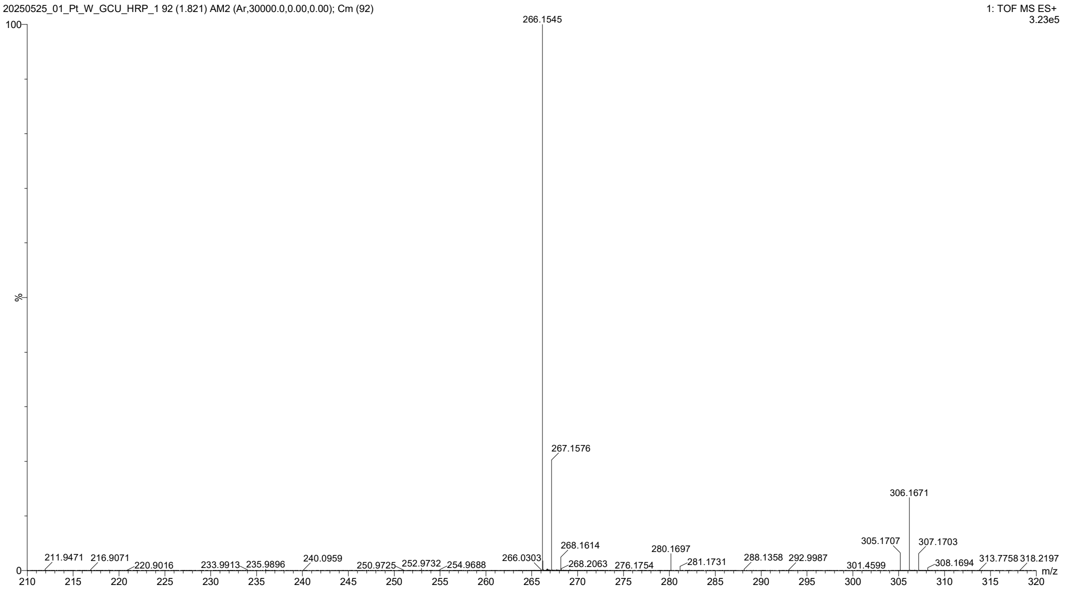

Figure S19. HR-MS of ((6R,8R)-7,7-dimethyl-5,6,7,8-tetrahydro-6,8-methanoisoquinolin-3-yl)phenol (**W**)

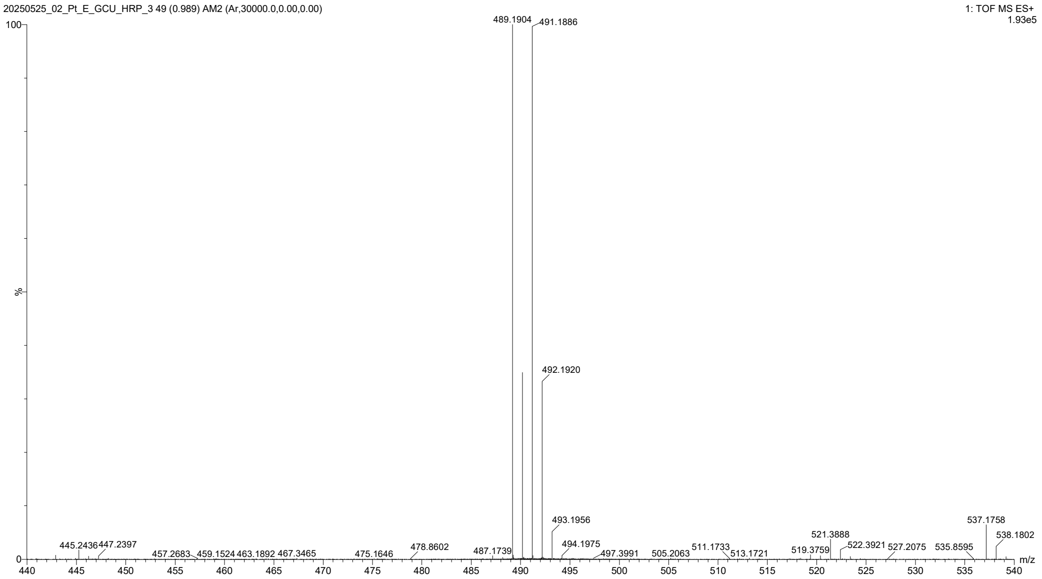

Figure S20. HR-MS of 2.2.3. 3-bromo-5-(4-(tert-butyl)pyridin-2-yl)-7,7,10,10-tetramethyl-7,8,9,10- tetrahydro-5H-benzo[b]carbazole (**E1**)

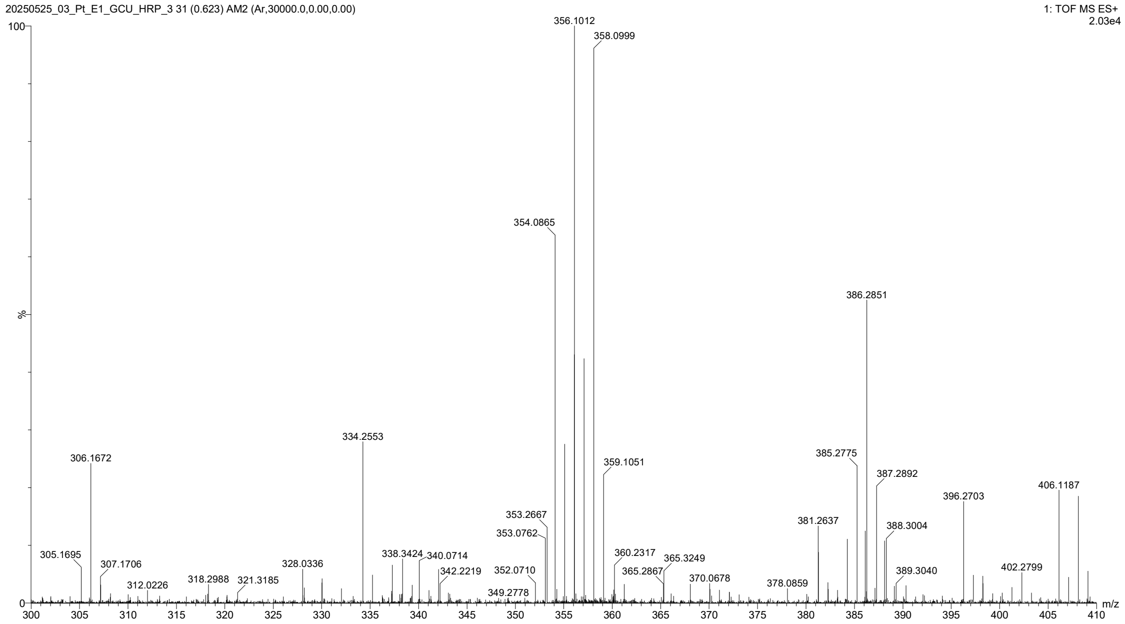

Figure S21. HR-MS of 3-bromo-5-(4-(tert-butyl)pyridin-2-yl)-7,7,10,10-tetramethyl-7,8,9,10- tetrahydro-5H-benzo[b]carbazole (**E**)

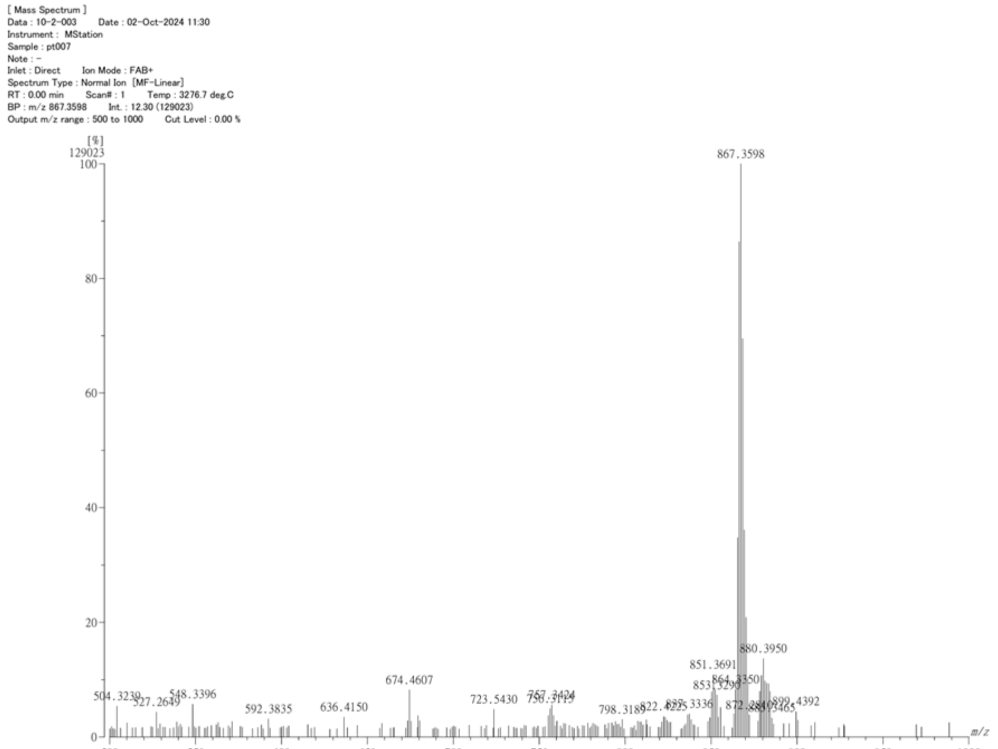

Figure S22. HR-MS of PhPiPy-O-PytmCz (P1)

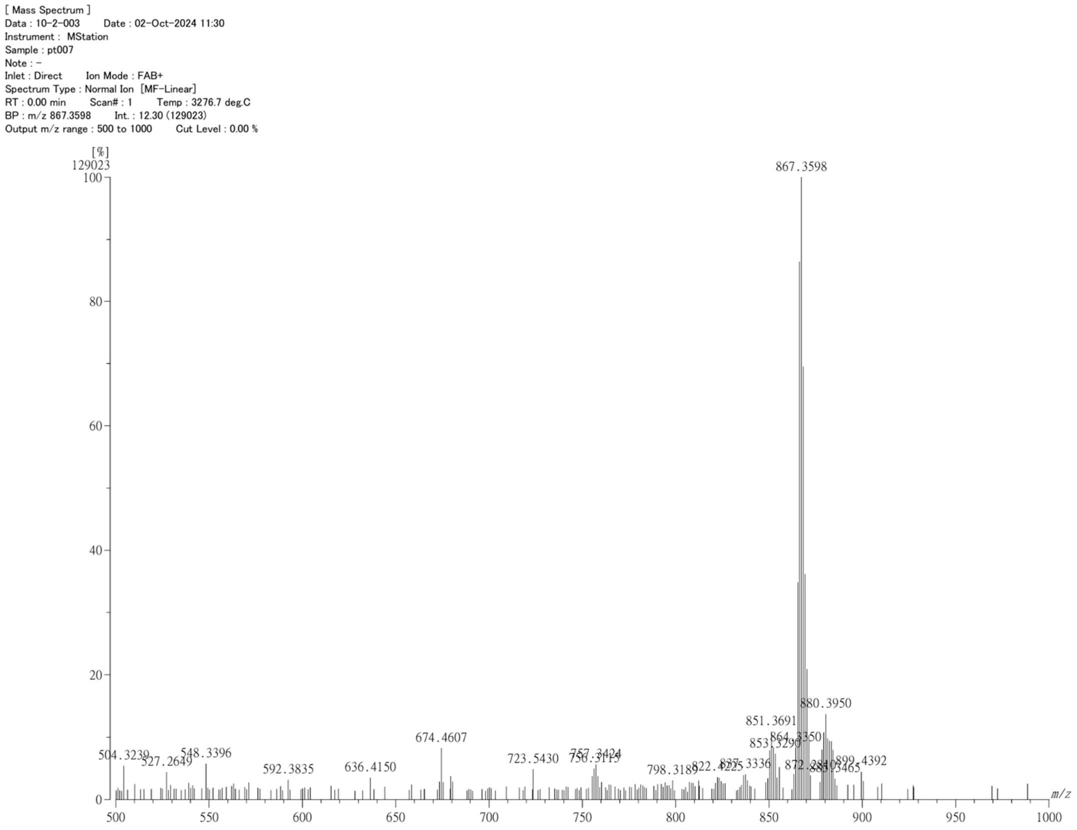

Figure S23. HR-MS of Pt(PhPiPy-O-PytmCz).

Table S1. Computed absorption wavelengths ($\lambda_{calc.}$ in nm) and oscillator strengths ($f_{calc.}$) for Pt(PhPiPy-O-PytmCz) from TD-DFT calculations using the B3LYP/LANL2DZ geometries at the first triplet excited state (T1) optimized geometries.

state	λcalc (/nm)	fcalc	Major contribution
		Pt007 T1	
1		0.0109	HOMO -> LUMO (32.62%)-alpha MO
			HOMO -> LUMO (93.35%)-beta MO
2		0.0015	HOMO -> LUMO+3 (12.64%)
			HOMO-3 -> LUMO (19.89%)
			HOMO-2 -> LUMO (11.59%)
			HOMO-1 -> LUMO (94.71%)
3		0.0288	HOMO -> LUMO (93.81%)-alpha MO
			HOMO -> LUMO (30.99%)-beta MO

Table S2. Molecular orbital energies (in eV) and molecular orbital distributions (in %) of Pt(PhPiPy-O-PytmCz) at the first triplet excited state (T1) optimized geometries.

	E(eV)	Pt	myPyPh	O	tmCz	tbuPyridine
			Pt007 T1			
LUMO+3	-0.79	2	24	0	22	51
LUMO+2	-0.82	5	37	0	20	38
LUMO+1	-1.3	3	6	0	9	82
LUMO	-3.77	24	48	9	18	2
HOMO	-2.43	5	89	5	3	3
HOMO-1	-5.22	10	20	10	58	1
HOMO-2	-5.43	13	10	13	66	11
HOMO-3	-5.46	8	88	8	3	1

Table S3. Cartesian coordinates of the ground state (S0) fully optimized geometry of Pt(PhPiPy-O-PytmCz) from TD-DFT calculations.

atom	X	Y	Z												
C	-6.39853	-7.33335	-1.08952	N	-1.76299	-1.39939	0.98291	C	3.26059	-6.10834	2.05948	H	-6.38913	2.43146	-0.82173
C	-4.90233	-7.36023	-0.64914	C	-2.97561	-1.98927	1.07416	C	1.95249	-6.45264	1.72085	H	-3.06498	1.60112	0.24268
C	-4.66661	-8.18101	0.65374	C	-4.16612	-1.40425	0.73509	H	-6.69040	-8.31676	-1.51049	H	1.64756	3.73886	3.54266
H	-5.25818	-9.10769	0.81018	C	-4.18431	-0.09232	0.32705	H	-6.54491	-6.55406	-1.86851	H	1.82274	6.45671	3.45828
C	-5.06653	-6.90089	1.43729	C	-5.52325	0.53356	-0.12397	H	-7.12719	-7.10165	-0.28872	H	0.69643	7.73321	4.05725
C	-3.13139	-8.42586	0.92622	C	-6.00583	-0.21086	-1.38791	H	-4.60868	-6.70114	2.43386	H	0.69603	7.24449	2.32177
C	-4.04743	-7.65787	-1.89637	C	-6.58197	0.39971	0.99439	H	-6.16644	-6.81264	1.54196	H	1.01608	4.92650	5.29228
C	-4.55810	-6.12090	0.19465	C	-5.41133	2.03208	-0.47597	H	-2.72346	-9.18022	0.22226	H	-0.72230	4.48533	5.40338
H	-5.04070	-5.14815	-0.05031	C	-2.96743	0.60057	0.43695	H	-3.02822	-8.81634	1.96142	H	-0.15971	6.12990	5.87481
C	-3.05783	-5.99473	0.43201	C	-1.73757	-0.02660	0.82594	H	-4.33240	-8.64594	-2.31405	H	-1.78947	7.22602	4.39275
C	-2.34790	-7.12071	0.82973	N	-0.56008	0.75697	1.22359	H	-2.96126	-7.67266	-1.68985	H	-2.42928	5.58668	4.03874
C	-0.98885	-6.98626	1.13043	C	-0.45821	2.12535	1.44072	H	-4.22861	-6.87669	-2.66562	H	-1.40367	7.58328	1.98422
C	-2.43337	-4.77010	0.38063	C	0.63863	2.40594	2.21000	H	-0.42819	-7.86853	1.39974	H	-3.10473	7.32024	2.32132
N	-1.17048	-4.57472	0.89278	C	0.82694	3.63277	2.84834	H	-3.04497	-4.01067	-0.04595	H	-2.42693	6.96467	-0.39826
C	-0.41383	-5.68600	1.16757	C	-0.17823	4.62488	2.71592	H	4.15085	-0.39848	3.15355	H	-1.76331	5.34823	-0.85294
C	0.99458	-5.44158	1.52113	C	-0.25365	5.80213	3.69307	H	3.19958	1.87192	3.25683	H	-0.71510	6.55164	-0.03482
C	1.35952	-4.09854	1.64799	C	0.79963	6.87292	3.36008	H	-3.04303	-2.92589	1.55323	H	-4.31080	5.90071	0.96593
C	2.65520	-3.75357	2.03541	C	-0.01548	5.30524	5.14423	H	-5.06776	-1.99603	0.81010	H	-3.93658	4.59963	2.16659
O	3.04168	-2.49128	2.29892	C	-1.70865	6.36683	3.69266	H	-6.15578	-1.29356	-1.19115	H	-3.73129	4.35890	0.38738
C	2.39488	-1.30258	2.27440	C	-2.11920	6.80258	2.29965	H	-5.25609	-0.11076	-2.20347	H	-2.01501	3.15608	0.33101
C	3.14991	-0.22524	2.77554	C	-2.15371	5.60056	1.31116	H	-6.97151	0.20301	-1.74892	H	4.61715	-4.51354	2.52101
C	2.62423	1.05785	2.83490	C	-1.75069	6.14263	-0.07835	H	-6.78544	-0.65890	1.25414	H	4.00262	-6.88198	2.21243
C	1.37325	1.26156	2.27986	C	-3.61048	5.07628	1.22095	H	-7.54853	0.84431	0.67338	H	1.71183	-7.49967	1.61433
C	0.60398	0.24636	1.73388	C	-1.16594	4.46579	1.70082	H	-6.24164	0.92347	1.91351				
C	1.05565	-1.09285	1.81262	C	-1.27604	3.21059	1.06688	H	-4.67237	2.20158	-1.28922				
Pt	-0.17014	-2.73063	1.31929	C	3.60619	-4.76681	2.22542	H	-5.11931	2.60858	0.42433				
